# VXX-401, a novel anti-PCSK9 vaccine, reduces LDL-C in cynomolgus monkeys

**DOI:** 10.1016/j.jlr.2024.100497

**Published:** 2024-01-10

**Authors:** Madeline M. Vroom, Hanxin Lu, Maggie Lewis, Brett A. Thibodeaux, Jeanne K. Brooks, Matthew S. Longo, Martina M. Ramos, Jaya Sahni, Jonathan Wiggins, Justin D. Boyd, Shixia Wang, Shuang Ding, Michael Hellerstein, Valorie Ryan, Peter Powchik, Jean-Cosme Dodart

**Affiliations:** Vaxxinity, Inc, Dallas, TX, USA

**Keywords:** atherosclerosis, atherosclerotic cardiovascular disease, PCSK9 vaccine, hyperlipidemia, PCSK9 inhibitor, hypercholesterolemia

## Abstract

Atherosclerotic cardiovascular disease (ASCVD) remains the leading cause of disease burden in the world and is highly correlated with chronic elevations of LDL-C. LDL-C-lowering drugs, such as statins or monoclonal antibodies against proprotein convertase subtilisin/kexin type 9 (PCSK9), are known to reduce the risk of cardiovascular diseases; however, statins are associated with limited efficacy and poor adherence to treatment, whereas PCSK9 inhibitors are only prescribed to a “high-risk” patient population or those who have failed other therapies. Based on the proven efficacy and safety profile of existing monoclonal antibodies, we have developed a peptide-based vaccine against PCSK9, VXX-401, as an alternative option to treat hypercholesterolemia and prevent ASCVD. VXX-401 is designed to trigger a safe humoral immune response against PCSK9, resulting in the production of endogenous antibodies and a subsequent 30–40% reduction in blood LDL-C. In this article, VXX-401 demonstrates robust immunogenicity and sustained serum LDL-C-lowering effects in nonhuman primates. In addition, antibodies induced by VXX-401 bind to human PCSK9 with high affinity and block the inhibitory effect of PCSK9 on LDL-C uptake in a hepatic cell model. A repeat-dose toxicity study conducted in nonhuman primates under good laboratory practices toxicity indicated a suitable safety and tolerability profile, with injection site reactions being the main findings. As a promising safe and effective LDL-C-lowering therapy, VXX-401 may represent a broadly accessible and convenient option to treat hypercholesterolemia and prevent ASCVD.

Despite existing therapies that reduce the risk of cardiovascular events and death, cardiovascular disease is the leading cause of mortality worldwide. In 2019, cardiovascular disease was estimated to claim 18.6 million lives, amounting to 31% of all deaths recorded that year (1). The pathophysiological processes that potentiate heart failure are numerous and diverse. However, myocardial infarctions (i.e., heart attacks) and strokes account for 85% of all cardiovascular-related fatalities, of which the most significant cause is atherosclerotic cardiovascular disease (ASCVD). Atherosclerosis directly stems from the accumulation of LDL-C within the subendothelial space (i.e., intima) of the arteries. Specifically, sustained elevations in circulating LDL-C lead to the formation of lipid-laden plaques in and on the arterial walls, which are a major etiological factor of arterial blockage (2). Over time, the core of the plaque steadily expands because of the combined effects of cellular localization, lipid accumulation, as well as the buildup of necrotic and apoptotic debris (3, 4, 5). As the lesion grows, it becomes increasingly unstable and the walls of the blood vessel narrow, impeding blood flow. Plaque destabilization subsequently gives way to rupture, thrombosis, and potentially fatal cardiovascular distress.

Given its critical role in ASCVD, therapies that lower LDL-C have long been used by physicians for the treatment of patients with hypercholesterolemia, which affects more than 40% of the Western population. Statins, which inhibit cholesterol synthesis and promote its reabsorption, are considered standard of care. However, statin treatment is associated with high rates of noncompliance and discontinuation (6). Alternatively, inhibitors of proprotein convertase subtilisin/kexin type 9 (PCSK9) have demonstrated marked LDL-C-lowering properties but are only prescribed as second-line therapies and are accessible by only a subset of patients in need (7). PCSK9 is a secreted serine endoprotease that plays a critical role modulating lipoprotein homeostasis and non-HDL metabolism (8), in part by modulating the expression of LDL receptors (LDLRs) at the surface of hepatic cells (Fig. 1A). PCSK9-targeted therapies include the monoclonal antibodies (mAbs) evolocumab and alirocumab, as well as the long-acting, synthetic, small-interfering RNA (RNA interference) inclisiran. By inhibiting PCSK9 activity or production, these treatments have been shown to dramatically reduce LDL-C by 50–70% and attenuate the risk associated with ASCVD (9). Apart from the marked reduction in LDL-C and improvement in cardiovascular outcomes, PCSK9 inhibitors have an excellent safety profile and are generally well tolerated. In addition, clinical trials with PCSK9 inhibitors revealed neither treatment-related increase in the risk of new-onset diabetes nor the exacerbation of pre-existing glycemia, both of which are known, undesirable, side effects of statin medications (10, 11, 12, 13, 14). Although PCSK9 inhibitors have undeniably strengthened the modern-day armament of lipid-lowering therapies, their widespread adoption has been severely limited by cost (15). In fact, when evolocumab and alirocumab were approved by the Food and Drug Administration in 2015, their use was deemed to be cost effective only for patients at high risk of developing ASCVD. The RNA interference therapy, inclisiran, faces similar barriers to common use. Given that ASCVD remains the leading cause of disease burden in the world and that elevated levels of LDL-C are the primary risk factor for atherosclerosis, there is an urgent need to bridge the gap between highly effective anti-PCSK9 therapies and affordable, convenient, prevention options for patients.

**Fig. 1:**
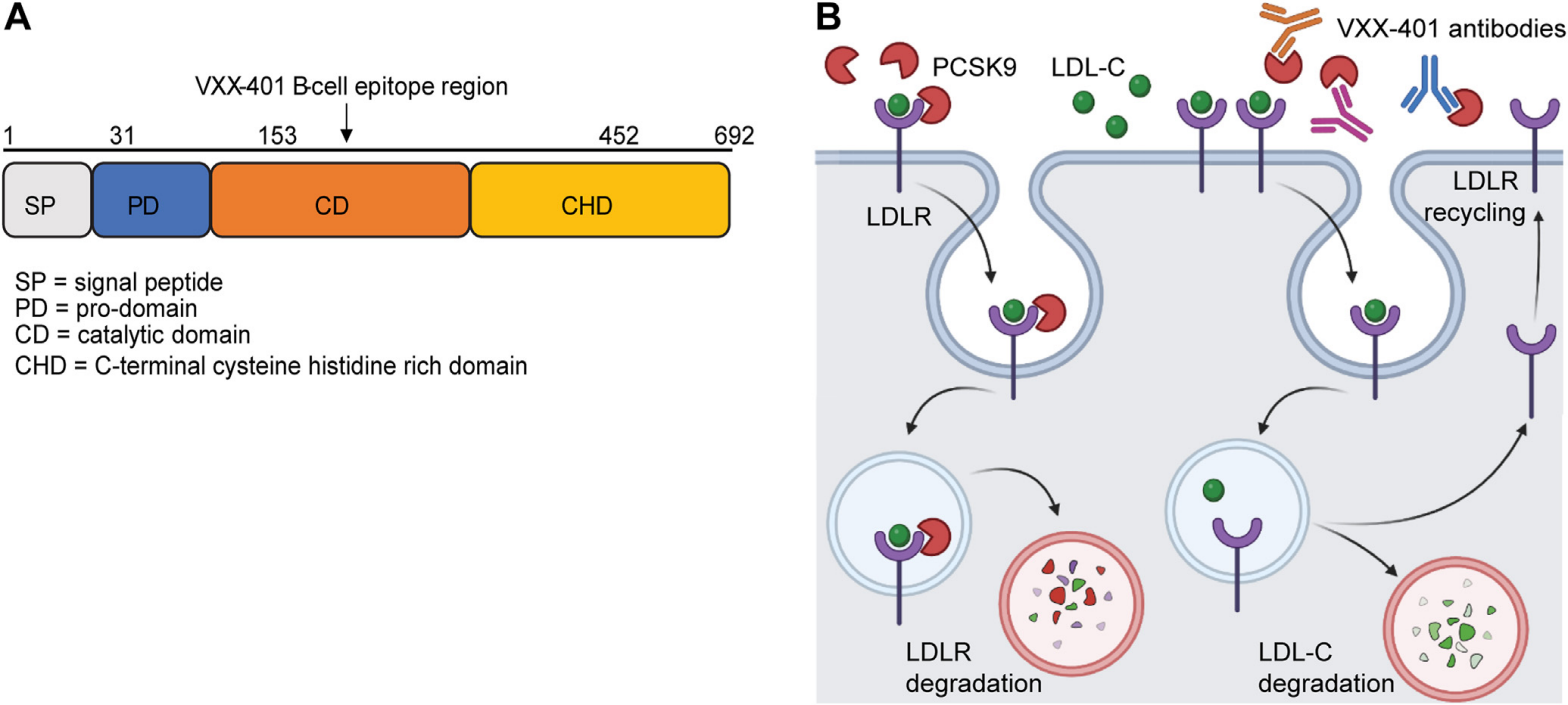
The cholesterol-lowering action of VXX-401. A: Unprocessed human PCSK9 harbors three distinct regions plus an N-terminal signal peptide. The black arrow indicates the location of p5494a, the B-cell epitope of VXX-401, in the catalytic domain. B: LDL-C is removed from circulation by hepatic uptake via LDLR. After binding, the LDL-LDLR complex is internalized and delivered to the endosome. In the absence of PCSK9, the acidic pH induces dissociation. The receptor is recycled back to the cell surface for reuse, whereas the LDL-C is broken down in the late endosome and lysosome. When PCSKS9 is present, it binds to the receptor and is internalized alongside the complex, preventing the endosomal dissociation of LDLR from LDL-C. Thus, the receptor is degraded along with LDL-C. Reductions in cell surface-associated LDLR blunt the liver’s ability to absorb LDL-C, leading to an increase that may trigger atherosclerosis. By binding to PCSK9, VXX-401 antibodies prevent the hepatic degradation of LDLR and promote endocytic recycling, thus preserving LDL-C absorption. Diagram created with BioRender.com.

Toward that end, Vaxxinity has developed VXX-401, a novel anti-PCSK9 vaccine for the treatment of hypercholesterolemia and long-term prevention of ASCVD. The active ingredient in VXX-401 is a fully synthetic peptide comprised of a proprietary T-helper peptide linked to a B-cell epitope designed to induce antibodies against the catalytic domain of human PCSK9 (Fig. 1B). The B-cell epitope alone is referred to as p5494a, whereas the full peptide immunogen is designated p5494kb. VXX-401 is the fully formulated vaccine, consisting of p5494kb as well as adjuvants. In this article, we describe the outcomes of three preclinical studies investigating the pharmacological effects of p5494kb and VXX-401 vaccination in nonhuman primates (NHPs). The results highlighted herein provide a compelling testament to the clinical potential of VXX-401 as an effective vaccine that lowers LDL-C and, in doing so, stands to mitigate the risk associated with ASCVD.

## Materials and methods

### Animal studies

Guinea pig studies were conducted at AlphaPreclinical, whereas NHP studies were conducted at Biomere, Envol, or IITRI. All study designs and animal protocols were approved by the governing Institutional Animal Care and Use Committee of the respective institutions. In a preliminary study, peptides targeting functionally distinct regions of PCSK9 were screened for immunogenicity (data not shown). Confirmation of the immunogenicity of the top five peptides of interest was assessed in male Duncan-Hartley guinea pigs (n = 5 per group). Animals were immunized intramuscularly (IM) on weeks 0 (400 μg of peptide with AdjuPhos and CpG), 3, 6, 9, and 12 (100 μg of peptide with AdjuPhos and CpG).

A follow-up study evaluated the immunogenicity of p5494kb, the peptide with the highest immunogenicity, formulated with different adjuvants. This included the alum-based adjuvants AdjuPhos and Alhydrogel, CpG1 and CpG3 (oligonucleotides of different lengths), as well as MF59 and ISA 51 VG, which are oil-in-water emulsions. These adjuvants are often required to induce a strong immune response and might activate different types of immune responses, which are also dependent on the type of immunogen they contain (16, 17, 18). The guinea pigs (n = 3 per group) received IM injections on week 0 (400 μg of p5494kb in adjuvants) as well as on weeks 3 and 6 (100 μg of p5494kb in adjuvants). Blood samples were collected before each injection and at the terminal time point of week 9. Formulation was further optimized in a third study that evaluated the effects of escalating doses of CpG1 on the immunogenicity of p5494kb. In this study, p5494kb (300 μg/dose) was formulated in AdjuPhos and increasing amounts of CpG1 per dose (0, 25, 100, 400, or 1,600 μg). Guinea pigs (n = 5 per group) received IM injections of placebo (saline) or p5494kb at weeks 0 and 2; serum samples were collected before each dose and at the terminal time point of week 4.

The initial pilot/proof-of-concept study in NHPs evaluated the tolerability, immunogenicity, and pharmacodynamics of p5494kb in non-naïve male cynomolgus monkeys between 2 and 4 years old and weighing 2–4 kg. This study was conducted prior to formulation optimization in guinea pigs and assessed the effects of a priming regimen of p5494kb formulated in AdjuPhos (0.8 mg/dose) and CpG3 (50 μg/dose), followed by a boosting regimen of p5494kb formulated in ISA51 VG (50 μg/dose) and CpG3 (50 μg/dose). Monkeys were randomized across study groups based on predose serum levels of LDL-C. Each animal (n = 3 group) received a total of six IM injections (0.5 ml/dose) of 300 μg/dose p5494kb or adjuvant placebo. The priming regimen consisted of a 300 μg p5494kb given IM on weeks 0, 3, and 6. The boosting regimen consisted of a 300 μg p5494kb given IM on weeks 13, 16, and 19. A separate group of cynomolgus monkeys (n = 3) received IM injections of saline on weeks 0, 3, 6, 13, and 16 and received a single intravenous bolus of evolocumab (3 mg/kg; Amgen) on week 19. Animals were followed through week 25, with blood samples collected every week until completion.

After formulation optimization, escalating doses of VXX-401 (p5494kb in AdjuPhos and CpG1) were evaluated for immunogenicity and efficacy in a separate cohort of naïve Cynomolgus monkeys. Monkeys were randomly assigned to seven groups (n = 3 per group) and received IM injections (0.5 ml/dose) of placebo or VXX-401 (10, 30, 100, 300, and 900 μg/dose) on weeks 0, 3, and 6 as the priming regimen. At week 24, all animals received a placebo or booster dose of VXX-401 (100 μg/dose). Injection sites were monitored at 2, 24, and 48 h after each dose; body weight was measured prior to each dose and every 2 weeks thereafter; and body temperature was measured predose and 24 h after each injection. Blood samples were collected to evaluate clinical chemistry, including LDL-C, HDL-C, and total cholesterol (predose and weekly); hematology (predose, day 42, and day 168); cytokines (6 h postdosing on days 0, 21, and 42; Supplemental Dataset 1); and serum anti-PCSK9 titers (predose and days 0, 21, 42, 63, 84, 105, 126, 147,168, and 189).

Finally, VXX-401 was evaluated in a third cohort of cynomolgus monkeys to assess safety in a repeat-dose toxicity study under good laboratory practices (GLPs). About 37 naïve monkeys (n = 9–10 per group) were randomized to receive either saline placebo, adjuvant control (0.8 mg AdjuPhos and 100 μg CpG1), a low dose of VXX-401 (100 μg peptide/dose), or a high dose of VXX-401 (600 μg peptide/dose) administered IM (0.5 ml/dose) for five total injections on weeks 0, 3, 6, 9, and 12. Terminal necropsy was performed at day 88, and a standard panel of tissues was collected for histopathological evaluation. Animals were between 2 and 4 years old and weighed between 2.7–4.4 kg for males (n = 19, 4–5 per group) or 2.3–5.2 kg for females (n = 18, 4–5 per group) at the first dose. Clinical observations made over the course of the study included mortality/moribundity; physical examinations; body weight; body temperature; food consumption; and evaluation of injection sites for reactogenicity. Safety pharmacology assessments for the cardiovascular, respiratory, and central nervous system were also included. In addition, blood and urine samples were collected for clinical pathology evaluations, including hematology, coagulation, clinical chemistry (i.e., LDL, HDL, total cholesterol, and triglycerides), and urinalysis. Serum samples were obtained for evaluation of anti-PCSK9 antibody titers and cytokines. The summarized findings of the GLP toxicity study can be found in Supplemental Dataset 2.

### ELISAs

Anti-PCSK9 and T-helper peptide titers were determined by ELISA. High-bind 96-well microplates were coated with 2 μg/ml of human recombinant PCSK9 (Kemp Proteins) or T-helper peptide in 1× PBS and incubated overnight at 2–8°C. Coated plates were blocked in PBS SuperBlock™ Buffer (Thermo Scientific) for 2 h at 28°C with shaking at 500 rpm. Serum samples were serially diluted in half-log iterations from an initial dilution factor of 20 in 1× PBS containing 2% BSA in a 12-point series, then arrayed in duplicate into assay plates, and incubated for 1 h at 28°C. HRP-conjugated Protein A/G (Thermo Scientific) was used for antibody detection at a 1:20,000 dilution in PBS-T (PBS with 0.05% Tween and 2% Difco skim milk). After incubation at 28°C, 3,3′,5,5′-tetramethylbenzidine (TMB) Ultra ELISA substrate (Thermo Scientific) was applied to each well, and the reaction was stopped after 5 min by addition of 2 N H_2_SO_4_. Absorbance was measured at 450 nm on a SpectraMax iD5 plate reader (Molecular Devices) within 10 min of halting the reaction. A nonlinear four-parameter curve fit was applied to each dilution series to calculate the antibody titer, expressed as the half-maximal effective concentration (EC_50_; SoftMax Pro, version 7.1 or GraphPad Prism, version 9.2). For titers below the lower detection limit, a titer of <20 (starting dilution factor) was reported and a value of 10 assigned to the sample to calculate the group mean. Assay plates were washed four times between steps in 1× PBS-T (0.05%) except after blocking (1× wash) and secondary incubation (6× wash). All working volumes in the microplate were 100 μl per well, apart from the block buffer, which was 200 μl.

To measure PCSK9-specific IgG subtypes by ELISA in the repeat-dosing study, microplates were coated with 1 μg/ml PCSK9 recombinant protein, blocked with 10% BSA, and incubated with diluted serum samples from a 12-point series, starting with a dilution factor of 50. Rabbit-antimonkey IgG1, IgG2, IgG3, or IgG4 at a 1:5,000 dilution in 1× PBS with 2% BSA was subsequently applied to the plates, followed by incubation with mouse-anti-rabbit IgG-HRP conjugate at 1:10,000 dilution in 1× PBS with 2% BSA. Following TMB development for 10 min, the reciprocal IgG subclass titers were determined as the EC_50_ from a nonlinear four-parameter curve. For titers below the lower detection limit, a titer of <50 (starting dilution factor) was reported and a value of 25 assigned to the sample to calculate the group mean. All procedural details not explicitly reported are the same as the anti-PCSK9 ELISA described previously.

### LDL and HDL quantification

For lipid analysis of NHP serum samples, LDL and HDL-C were directly measured on an IDEXX ProCyte or a Vet Axcel chemistry analyzer. For analysis and visualization, LDL-C and HDL-C levels were averaged every two (pilot study) to three (dose-ranging and GLP toxicity study) consecutive weeks, and normalized to the predose level, with comparisons made to the placebo and/or adjuvant controls. The results are presented herein as change-from-baseline, which was calculated at the percentage of cholesterol measured at a given time point relative to the predose level for each individual monkey. The baseline LDL-C ranges for the pilot, the dose-range finding, and GLP toxicity studies in NHP were 27–75 mg/dl, 23–143 mg/dl, and 28.1–89 mg/dl, respectively.

### Total IgG isolation and concentration from experimental hyperimmune sera

For antibody characterization, sera collected from immunized monkeys (GLP toxicity study, terminal bleed) were pooled and passed through a 0.22 μm filter to remove particulate matter. All materials and reagents were adjusted to room temperature prior to use. Washes and elution were accomplished via centrifugation for 1 min at 1,000 *g* (Eppendorf). To isolate total IgG fraction, NAb A/G protein columns (Thermo Scientific) were washed with three resin volumes of Pierce Protein A/G Binding Buffer (Thermo Scientific). Sera filtrate was diluted two-fold in binding buffer and then applied to the column and incubated with end-over-end mixing for 2 h. The resin was subsequently washed with three volumes of binding buffer, and bound antibody was eluted four to five times in 5 ml of IgG Elution Buffer (Thermo Scientific) and neutralized with 250 μl of Tris-HCl (pH 8.5; Anatrace). Triplicate IgG isolations were performed for each group. Total IgG fraction was concentrated and buffer exchanged into ultrapure DNase/RNase-free distilled water (Invitrogen) via 100 kDa Amicon filter units (MilliporeSigma) with centrifugation at 6°C and 4,200 *g*. The protein content of the total IgG fraction concentrate was subsequently determined via QuBit Fluorometer 4.0 (Invitrogen) protein assay per the manufacturer’s instructions. Afterward, the IgG fraction concentrate was stored at −20°C until affinity purification.

### Affinity purification of antibodies against human PCSK9

Disposable columns were packed with 2 ml of streptavidin agarose slurry (Thermo Fisher Scientific) and stored in ultrapure deionized water (i.e., sterile and DNase-, RNase-, and protease free; Invitrogen) supplemented with sodium azide (G-Biosciences) to a final concentration of 0.02% at 2–8°C. All materials and reagents were adjusted to room temperature prior to use, and the washes and elution were accomplished via gravity flow. To link the biotinylated PCSK9 protein, streptavidin columns were washed with five resin bed volumes of 1× PBS (R&D). Recombinant human PCSK9 amounting to 2 mg of total protein was applied to each streptavidin column and incubated overnight at 2–8°C. The next day, each column was washed with five volumes of binding buffer. Total IgG fraction concentrate was diluted in binding buffer, applied to the column, and incubated in the resin bed overnight at 2–8°C. The next day, each column was washed with five volumes of binding buffer, and bound anti-PCSK9 antibody was eluted as previously described. Like fraction was pooled and concentrated in a 100 kDa Amicon filter unit (MilliporeSigma) with centrifugation at 6°C and 4,200 *g*, then buffer exchanged into ultrapure DNase/RNase-free deionized water (Invitrogen). The protein content of the affinity-purified antibody fraction was determined via QuBit Fluorometer 4.0 protein assay per the manufacturer’s instructions. Afterward, the anti-PCSK9 antibody was stored at −80°C.

### Antibody binding potency

The binding potency of the affinity-purified NHP antibodies was determined by ELISA as described previously for titers with two modifications. First, the VXX-401 antibody and evolocumab were normalized to a starting concentration of 1.44 mg/ml prior to serial dilutions and second, the TMB development time was 10 min.

### LDL uptake assay

The uptake of pHrodo-labeled LDL in HepG2 cells was performed using Invitrogen™Image-iT™ Low Density Lipoprotein Uptake Kit, pHrodo™ Red according to the manufacturer’s instruction with minor modifications. Cellular viability was assessed via acridine orange and propidium iodide staining via Cellaca MX (Nexcelom), after which DMEM HepG2 suspensions were plated at 40,000 cells/well in a black-welled, clear-bottomed, 96-well imaging plate (Krystal) and allowed to adhere for at least 5 h at 37°C in 5% CO_2_ and 95% humidity. Commercial and NHP affinity-purified anti-PCSK9 antibodies were serially diluted in starvation medium (DMEM, no phenol red with 1× Pen-Strep and l-glutamine) supplemented with 10 μg/ml PCSK9 and coincubated for 30 min at ambient temperature. During this step, DMEM was removed and the HepG2 monolayer was washed with starvation medium. After, the PCSK9-antibody solutions were added to the HepG2 plate and incubated overnight as previously described. For control purposes, medium-only and PCSK9 treatment groups without antibody were also included. The next day, the medium in the HepG2 assay plate was exchanged for DMEM starvation medium supplemented with LDL-pHrodo to a final concentration of 2.5 μg/ml. Images were collected via IncuCyte SX5 (Sartorius) every 30 min for 6 h, capturing 4 fields per well under normal phase conditions as well as with the orange, fluorescent channel (i.e., Cy5) at 10× magnification. Using the IncuCyte software, version 2021A, cells and pHrodo-LDL signal were segmented in phase and Cy5 channels, respectively. For cells, segmentation adjustment was set to 0.5 with a minimum area of 15 μm^2^. For pHrodo-LDL segmentation, Top-Hat method was selected with a radius of 100.00 μm, intensity threshold (optimized codon usage) of 0.50, and area range of 10–500 μm^2^. Cell area, cell confluence, LDL count, LDL total area, and LDL total integrated intensity were measured, then the LDL signal was calculated by dividing the total pHrodo-LDL area by cell area. The SX5 data at 4 h were analyzed in GraphPad Prism, version 9.2 (GraphPad Software, Inc) to calculate percent of LDL-C uptake relative to the untreated control.

### Peripheral blood mononuclear cell collection and ELISpot cytokine secretion assays

For peripheral blood mononuclear cell (PBMC) collection from the GLP toxicity study, blood samples were collected prior to immunization (day −6) and postimmunization (day 56), placed in K_2_EDTA tubes, and centrifuged at 450 *g* for 5 min to separate plasma from blood cells. After centrifugation, plasma was separated into two aliquots (at least 1 ml each in sterile cryovials, flash frozen and stored at −80°C), and the remaining blood cells resuspended in three volumes of Plasma Lytic A (pH 7.4; Baxter), which is placed carefully on the top of two volumes of Ficoll-Paque Plus (Cytiva). The cells are centrifuged at 650 *g* for 1 h with the brake turned off. Harvested PBMCs were washed once with Plasma Lytic A and counted with acridine orange and propidium iodide fluorescent staining in Vision cellometer. Cells were then placed in the freezing medium consisting of 10% FBS and 6% DMSO at 5.10^6^/ml/vial, frozen in a controlled rate freezing apparatus, and stored under vapor phase liquid nitrogen conditions.

To measure PBMC responses, ELISpot assays were performed using commercially available NHP IFN-γ ELISpotPLUS kits and the human IL-4 T-cell ELISpot kits (Mabtech). The cryopreserved PBMCs were first recovered in RPMI containing 20% FBS and 1% Pen-Strep. Next, the viable cells were counted and plated at a density of 200,000 cells/well into ELISpot plates precoated with NHP IFN-γ or human IL-4 capture antibodies. The PBMCs were subsequently stimulated with the T-helper peptide, the PCSK9 B-cell epitope (i.e., p5494a), or the VXX-01 peptide (i.e., p5494kb). All treatments were administered at a final concentration of 5 μg/ml, including the positive mitogen controls PHA-L and PHA-P (i.e., phytohemagglutinin L and P). An additional subpopulation of PBMCs on the assay plate received no treatment (i.e., medium blanks) as a negative control. After incubation at 37°C with 5% CO_2_ and 95% humidity for 40–42 h, the IFN-γ and IL-4 plates were washed and incubated with biotinylated-anti-NHP IFN-γ or biotinylated-antihuman IL-4 at 1 μg/ml for 2 h. After another wash, the plates were incubated with HRP-streptavidin (1:1,000 dilution) for 2 h. Cytokine spots were developed with ready-to-use TMB substrate for 10–20 min per the manufacturer’s instructions, after which the plates were washed with water and air dried. Finally, the assay plates were scanned on an AID iSpot reader to quantify cytokine-secreting cells. The results, expressed as spot-forming units per million cells, were analyzed after subtracting values of the negative control cells from that of the treated cells.

### MesoScaleDiscovery cytokine profiles

Serum cytokines (IFN-γ, IL-1β, IL-2, IL-4, IL-6, IL-8, IL-13, IL-10, and TNF-α) were analyzed using the UPLEX NHP cytokine kit from Mesoscale Discovery according to the manufacturer’s instructions. MSD plates were read using the ESO QuickPlex SQ 120MM Reader, and data were processed and analyzed using the Discovery Workbench software, version 4.0. All standards and samples were analyzed in duplicate.

### Replicates, data processing, and statistical analyses

All samples were assayed in duplicate or triplicate unless otherwise noted. Lipid percentages were rounded to the nearest whole number. The fit of each dataset was tested via Anderson-Darling, D’Agostino-Pearson, Shapiro-Wilk, as well as Kolmogorov-Smirnov. Statistical significance was evaluated using Welch’s *t*-test if *P* >0.05 for both the treatment and control groups. Mann-Whitney-Wilcoxon *U* test was used in all other instances. For both parametric and nonparametric statistical analyses, the threshold for significance was set at *P* ≤0.05. Analyses were performed using GraphPad Prism, version 9.2.0.

## Results

### Peptide and adjuvant selection by immunogenicity screening in guinea pigs

The peptide immunogen was selected through an extensive preclinical screening and optimization campaign. Briefly, peptide immunogens were designed to induce antibodies against several regions of the catalytic domain of PCSK9 and were tested in vivo for immunogenicity (Fig. 1A). The initial screen in guinea pigs suggested that the peptide immunogen p5494kb demonstrated the strongest immunogenicity, which was confirmed in a follow-up study showing that p5494kb induces a robust sustained antibody response (titers log_10_EC_50_ of 3–4) beginning at week 3 after the first injection (supplemental Fig. S1A). p5495kb corresponds to residues 153–162 of human PCSK9, which exhibits 80% and 100% sequence homology to the endogenous PCSK9 of guinea pigs and cynomolgus monkeys, respectively.

Adjuvant selection was achieved by analyzing the titers induced by p5494kb in formulation with ISA 51 VG, AdjuPhos, or Alhydrogel supplemented with CpG1 or CpG3. AddaVax was also tested, though not in complement with CpG. ISA 51 VG was only included as a positive control, as it is known to be a potent adjuvant but is often associated with tolerability issues (17). AdjuPhos and Alhydrogel supplemented with CpG1 and CpG3 performed comparably well, inducing considerable titers by week 3 that were sustained through the study end point of week 9 (supplemental Fig. S1B). AddaVax, however, was associated with lower antibody titers. Based on these data and previous experience with other similar investigational peptide vaccines (19, 20), AdjuPhos with CpG1 was selected as the adjuvant for VXX-401 (supplemental Fig. S1B).

Finally, the optimal ratio of CpG1 to peptide in the formulation was empirically determined by testing p5494kb (300 μg per dose) in AdjuPhos supplemented with CpG1 to a final concentration between 0 and 1,600 μg per dose in guinea pigs. Optimal immunogenicity was observed when p5494kb was formulated in 1.6 mg/ml AdjuPhos and 200 μg/ml of CpG1 (supplemental Fig. S1C). Thus, VXX-401 is the drug product containing the full peptide immunogen in 1.6 mg/ml AdjuPhos and 200 μg/ml CpG1. After adjuvant optimization, additional pharmacological studies were conducted with VXX-401 in cynomolgus monkeys to examine immunogenicity, efficacy, tolerability, and safety.

### VXX-401 is highly immunogenic in cynomolgus monkeys and reduces LDL-C without altering HDL-C

**Fig. 2:**
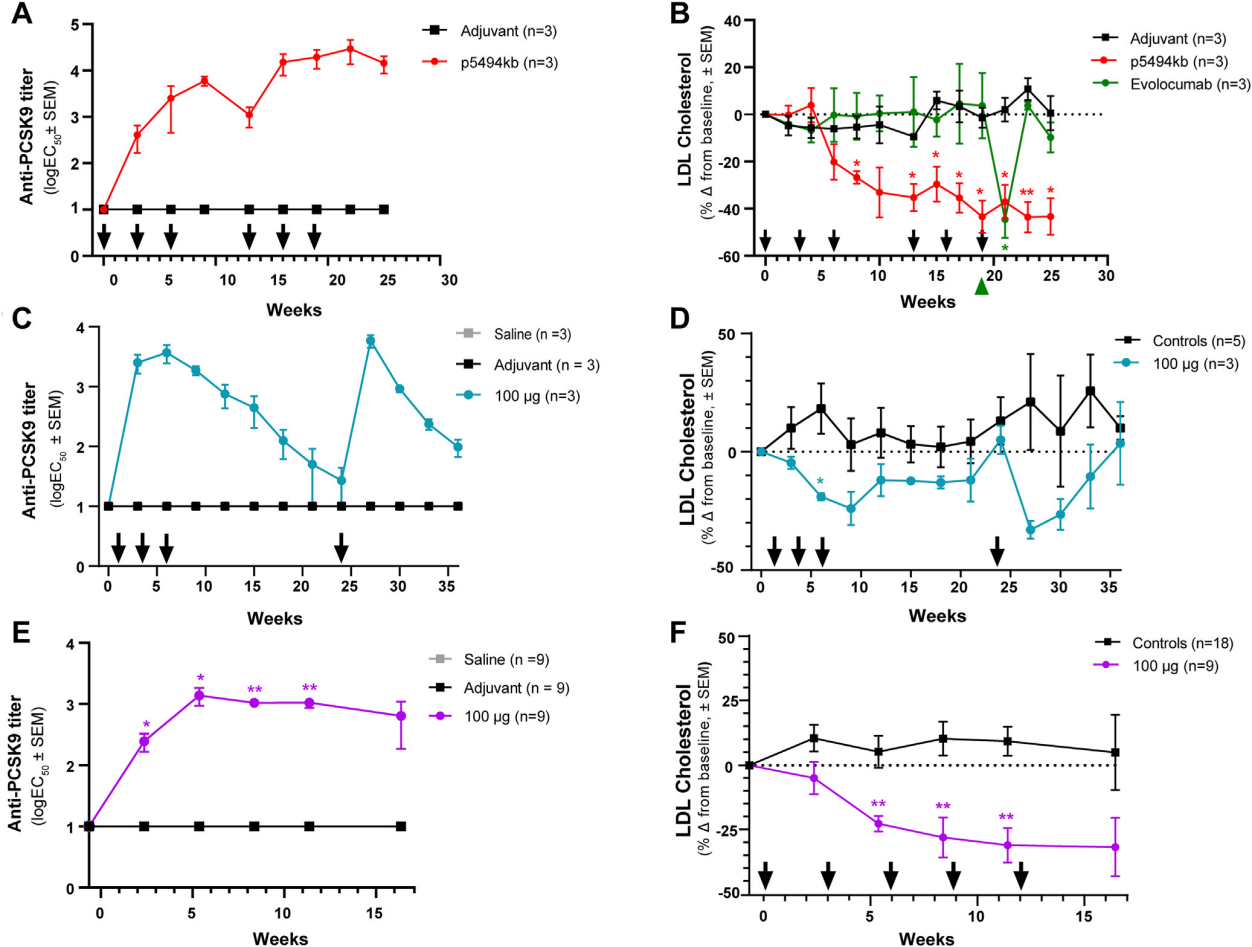
Vaccination against PCSK9 elicits a robust antibody response in cynomolgus monkeys and significantly reduces serum levels of LDL-C. A, B: Anti-PCSK9 titers and 2 week LDL-C metrics from the pilot study. The green triangle marks the time at which a comparator group received evolocumab. C, D: Anti-PCSK9 titers and 3 week LDL-C metrics from the dose-ranging study. E, F: Anti-PCSK9 titers and 3 week LDL-C averages in the GLP toxicity study. Arrows indicate the prime and boost schedule. All titers are expressed as the log_10_ value of the EC_50_. LDL-C is presented as percent change from baseline. Error bars represent the standard error of the mean. Asterisks denote a significant difference between treatment and control based on *P* ≤ 0.05 (∗) or *P* ≤ 0.01 (∗∗).

The immunogenicity and in vivo efficacy of p5494kb and VXX-401 were evaluated in NHPs, which show strong homology to human PCSK9 and also similar lipid metabolism compared with humans (21, 22). During the initial proof-of-concept study, p5494kb induced serum anti-PCSK9 antibodies with peak titers of log_10_EC_50_ of 3.75 by week 9. Titers thereafter decreased by one order of magnitude by week 13. A single boost of 300 μg p5494kb on week 13 restored antibody levels to log_10_EC_50_ 4.18 by week 16. Peak titers (log_10_EC_50_ of 4.47) were achieved after the three-dose boosting regimen by week 22 (Fig. 2A). As expected, no PCSK9-specific antibodies were detected in the sera from the adjuvant control group. In this study, the serum concentrations of LDL-C and HDL-C were quantified in 2-week intervals, and the average was normalized to the predose baseline. Serum LDL-C in the p5494kb treatment group progressively decreased over the course of the initial priming regimen, reaching a 35% reduction by week 13. After boosting, LDL-C further decreased to 43% from baseline, and this reduction was maintained through week 25 (Fig. 2B). At week 19, an intravenous injection of evolocumab was administered (3 mg/kg) to an additional group of monkeys that had previously received only saline injections. The bolus of mAb reduced serum LDL-C by a 2-week average of 45%, an extent that was statistically indistinguishable from that of the p5494kb treatment group. However, serum LDL-C was restored to baseline within 2 weeks of dosing with the mAb (Fig. 2B). None of the treatments were found to alter levels of serum HDL-C (supplemental Fig. S2A).

A follow-up study in a second cohort of cynomolgus monkeys was designed to evaluate the dose-dependent effects of VXX-401 on immunogenicity and serum LDL-C over a 48-week period. Anti-PCSK9 antibodies were detectable in serum within 3 weeks of the initial dose. At the 3 week time point, the highest titers (log_10_EC_50_ = 3.40) were observed in the monkeys dosed with 100 μg of VXX-401 (Fig. 2C). Overall, peak titers were achieved by week 6–9 and were comparable between the different dosage groups (i.e., log_10_EC_50_ 3.21–3.67). Antibody levels in all VXX-401 treatment groups exhibited a gradual decline beginning at week 12, with titers deteriorating to near baseline levels by week 24 (supplemental Fig. S3A). A boosting dose of 100 μg on week 24 restored maximal titers. In this study, LDL-C and HDL-C levels were quantified in 3 week intervals, and the average was normalized to the predose level. The most pronounced LDL-C reduction was observed in the animals that received 100 μg per dose of VXX401, which was decreased from baseline by 24% at week 9 (Fig. 2D). Lower than expected LDL reduction in this study might be explained by an experimental artifact. Indeed, the LDL-C in control animals increased during the first 6 weeks of treatment (up to 15% above baseline) suggesting that the lowering effects of VXX-401 on serum LDL-C may be underappreciated in this study. This fluctuation may be related to stress, changes in activity, or altered appetite, as the monkeys were transitioned from outdoor to indoor housing at the beginning of the study without an acclimation period. A comparison to control instead of baseline at week 6 would indeed suggest a 30–40% reduction in LDL-C. Animals that received 10 μg or 30 μg per dose of VXX-401 showed average LDL-C reductions between 10% and 15% from baseline. Finally, the monkeys given 300 μg or 900 μg of VXX-401 exhibited negligible reductions in LDL-C (<10%) compared with predose levels (supplemental Fig. S3B). The more pronounced effects of 100 μg VXX-401 on serum LDL-C supports the notion that a rapid antibody response might be necessary to overcome target-mediated clearance (23). Serum LDL-C returned to predose levels by week 24 in all groups, which coincided with the return of anti-PCSK9 titers to baseline. As expected, the single boost of 100 μg VXX-401 on week 24 reduced serum LDL-C by roughly 35% from the baseline taken right before the boost (Fig. 2D). Serum HDL-C was not altered by treatment in any of the groups (supplemental Fig. S2B).

Finally, the safety and tolerability of VXX-401 was evaluated under GLP conditions in NHPs. Consistent with previous studies, serum anti-PCSK9 antibody levels increased substantially between the initial priming dose and week 6, reaching a peak of log_10_EC_50_ of 3.14 in the 100 μg dose group (Fig. 2E). Antibody titers remained high and stable (i.e., log_10_EC_50_ of 3) from week 6 until the end of the study. No clear dose-dependency was observed between the 100 and 600 μg treatment groups (supplemental Fig. S3C). In addition, neither PCSK9-specific antibodies were detected in the adjuvant control nor the PBS placebo groups. In this study, VXX-401 treatment induced a sustained decrease in serum LDL-C, achieving up to 31% reduction from baseline (supplemental Fig. S3D and Fig. 2F). These changes are consistent with the expected pharmacological effects of VXX-401 and, as in the preceding two studies, no notable changes in HDL were observed (supplemental Fig. S2D). Total cholesterol and triglycerides also remained unchanged (Supplemental Dataset 2). In all three studies, antibody titers against the T-helper peptide component of VXX-401 were below the lower limit of quantification (data not shown).

### VXX-401 induces potent anti-PCSK9 antibodies and increases total PCSK9

**Fig. 3:**
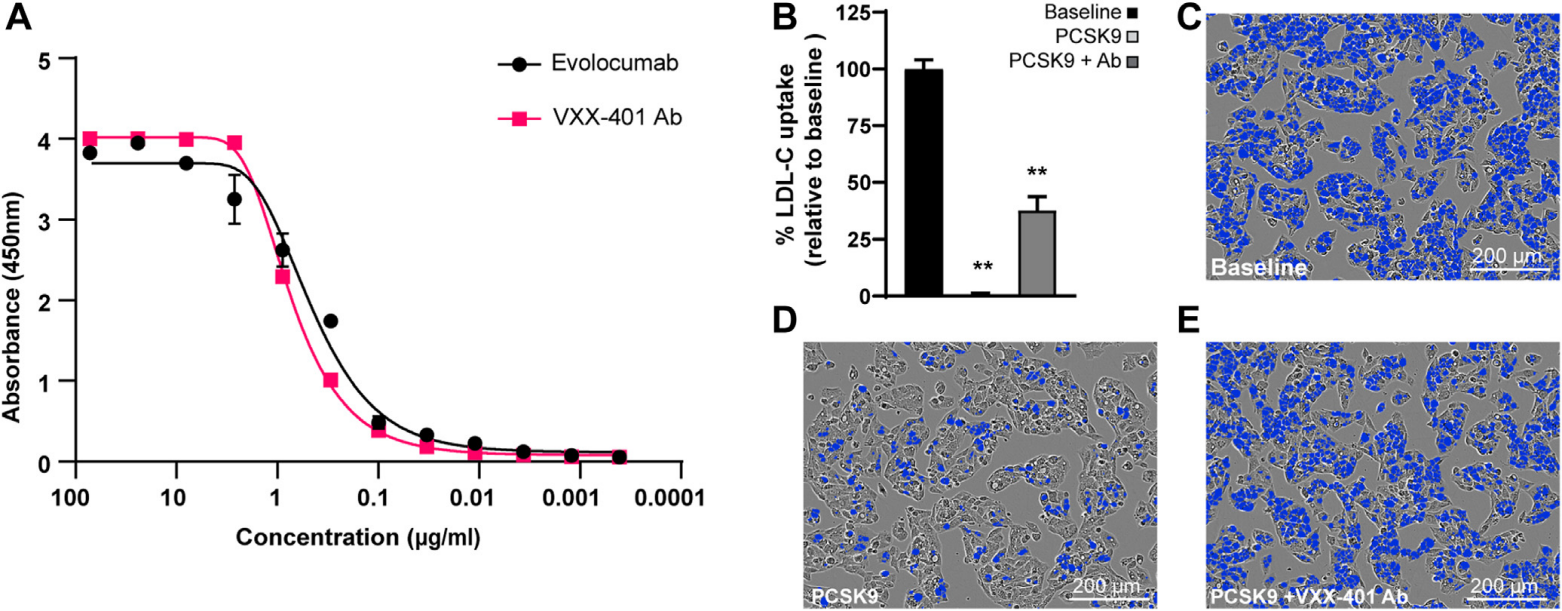
VXX-401 induces the production of highly potent and functionally active anti-PCSK9 antibodies. A: PCSK9 binding potency dose curve comparing affinity-purified antibodies from the 100 μg VXX-401 dosage group of the NHP GLP toxicity study to evolocumab. B: Bar graph showing uptake of pHrodo-conjugated LDL-C in HepG2 cells ± PCSK9 and the protective effect of affinity-purified VXX-401 antibodies. Uptake is expressed as the percent of baseline. C–E: SX5 images showing pHrodo-LDL-C uptake (blue mask) in HepG2 cells at baseline, with PCSK9 treatment, and rescue of uptake by VXX-401-derived antibodies. Error bars represent the standard error of the mean. Asterisks denote a significant difference between treatment and control based on *P* ≤ 0.05 (∗) or *P* ≤ 0.01 (∗∗).

Affinity-purified antibodies isolated from the sera of VXX-401-immunized NHP demonstrated potent binding to human PCSK9 via ELISA, with an EC_50_ of 0.621 μg/ml that was comparable to evolocumab at 0.547 μg/ml (Fig. 3A and Table 1). In addition, these antibodies had a protective effect on the uptake of pHrodo-labeled LDL in HepG2 cells, a human hepatocellular carcinoma line with endogenous expression of LDLR. More specifically, overnight incubation with exogenous recombinant human PCSK9 significantly reduced the uptake of pHrodo-labeled LDL-C to 31% of the control (Fig. 3B–D) as anticipated. Pretreatment of the HepG2 cultures with 100 μg/ml of affinity-purified anti-PCSK9 antibody, however, was sufficient to restore LDL uptake to 38% of its baseline value even in the presence of inhibitory PCSK9 (Fig. 3B, C, E). The potency and efficacy of VXX-401-induced antibodies were further compared with a therapeutic mAb against PCSK9 (Table 2). Total serum PCSK9 increased approximately 2-fold from baseline in the 100 and 600 μg dosage groups of the GLP toxicity study compared with the saline and adjuvant controls, which were consistent throughout (supplemental Fig. S4). Based on a nonlinear fit model for the dose-range study of anti-PCSK9 titers, the postprime antibody half-life was calculated to be 2.52 weeks, or approximately 17 days, which is comparable to that of evolocumab (11–17 days) (24).

**Table 1:** Binding potency of VXX-401 antibodies versus evolocumab

Antibody	EC_50_ (μg/ml)
NHP anti-PCSK9^a^	0.621
Evolocumab	0.547

a Affinity-purified antibodies from VXX-401-treated cynomolgus monkeys.

**Table 2:** Potency of VXX-401 antibodies and evolocumab in an LDL uptake assay

Antibody	EC_50_ (μg/ml)
NHP anti-PCSK9^a^	157.2
Evolocumab	6.00

a Affinity-purified antibodies from VXX-401-treated cynomolgus monkeys.

### VXX-401 induces a humoral response without cellular cytotoxicity or chronic inflammation

**Fig. 4:**
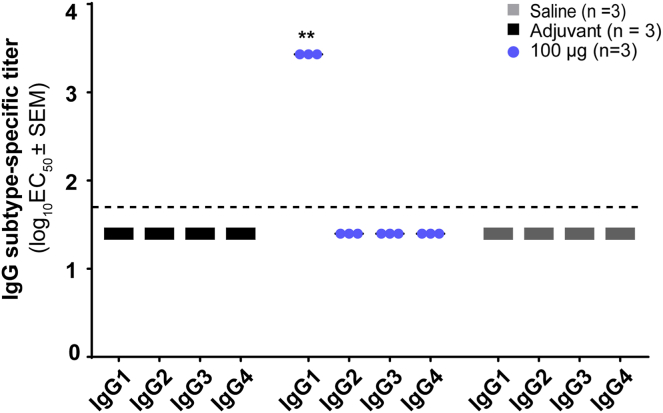
VXX-401 induces a humoral IgG_1_ immune response. IgG subtype-specific titers at week 9 for the saline placebo, adjuvant control, and 100 μg/dose treatment groups of the dose-ranging study. The dashed line indicates the lower limits of quantification for the subtype-specific ELISA titers. Asterisks denote a significant difference based on *P* ≤ 0.05 (∗) or *P* ≤ 0.01 (∗∗).

To characterize the type of immune response induced by VXX-401, we investigated the anti-PCSK9 antibody IgG subtypes, the response of PBMCs to various components of the peptide immunogen, as well as the serum cytokine profiles during immunization. Notably, VXX-401 was found to specifically evoke a robust immunoglobulin G, or IgG, antibody response. Subtyping analyses of the 100 μg dosage group at week 9 of the repeat-dosing NHP study revealed that the IgG produced by VXX-401 immunization were exclusively IgG_1_ (Fig. 4). IgG is among the most prevalent proteins in human serum, comprising up to 20% of total plasma protein. IgG can be further divided into four subclasses (IgG 1, 2, 3, and 4). IgG_1_ accounts for 60–65% of the IgG subclass and is predominantly responsible for the immune response against soluble proteins and peptide antigens (25). The results showing that VXX-401 elicited a IgG_1_-dominant response were therefore anticipated, given its nature as a synthetic peptide-based vaccine, and reveal a notable lack of proinflammatory subclasses (i.e., IgG_3_) and those with known pathogenic roles in autoimmune diseases (25, 26).

**Fig.5:**
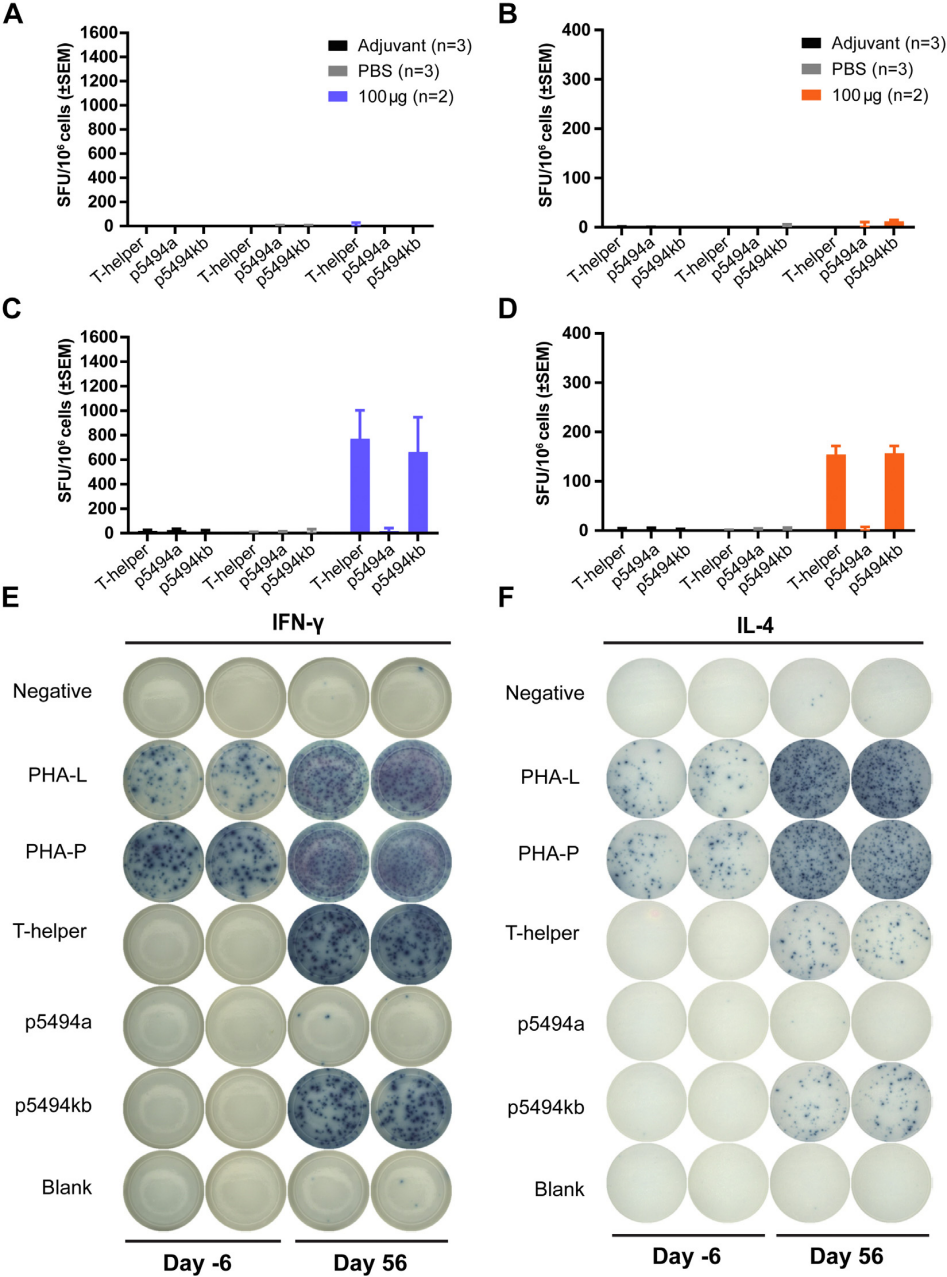
VXX-401 does not induce autoimmunity against endogenous PCSK9. A, B: Spot-forming units (SFUs) per million PBMC collected preimmunization. C, D: SFU per million PBMC collected postimmunization. E, F: Representative ELISpot scans for PBMC secretion of IFN-γ and IL-4 preimmunization and postimmunization. Error bars represent the standard error of the mean.

Further supporting the lack of an innate immune response was the absence of a proinflammatory cytokine response in naïve PBMCs stimulated with various components of the peptide immunogen. Indeed, PBMCs collected from NHPs prior to immunization with 100 μg VXX-401 did not release IFN-γ or IL-4 upon stimulation with any of the peptide components of p5494kb, as measured by ELISpot immunoassays (Fig. 5A, B). By contrast, PBMCs collected postimmunization released IFN-γ and IL-4 in response to the stimulation with the T-helper peptide alone or the full synthetic peptide immunogen (supplemental Fig. S5). Importantly, the PCSK9 B-cell epitope alone did not induce a cytokine response in PBMCs collected after immunization, indicating that endogenous PCSK9 will not be recognized as an antigen by the immune system. The latter observation is consistent with the limited duration of the antibody titers seen in vivo, which wane in the absence of re-exposure to the peptide immunogen. As anticipated, the PBMCs collected preimmunization and postimmunization showed a strong cytokine response to the PHA-positive controls, and minimal signal was observed in the unstimulated controls (Fig. 5E, F), which was subtracted as baseline.

Finally, the in vivo cytokine response profile to VXX-401 was evaluated preimmunization and postimmunization. Serum concentrations of IFN-γ, IL-1β, IL-2, IL-4, IL-6, IL-8, IL-13, IL-10, and TNF-α were quantified 2 h before and 6 h after the first, second, third, and fourth injections in the dose-ranging study in cynomolgus monkeys (Table 3). In Table 3, the data are expressed as log2 fold change (FC) between predose versus postdose. VXX-401 did not induce any change in serum concentrations of IFN-γ, IL-2, IL-4, IL-13, IL-10, and TNF-α across all conditions examined. A marginal decrease in serum IL-1β and IL-8 was observed in most dosing groups relative to their baseline values (log_2_ FC values < −1.0). In contrast, a slight increase in serum IL-6 was observed between predose and postdose measurements. Similar findings were obtained in the GLP toxicity study, where a significant increase (*P* < 0.01) in circulating levels of IL-6 in the 600 μg treatment group as compared with the adjuvant at weeks 3, 6, and 9. However, this increase was modest (log_2_ FC values < ±2), and there was no significant difference when comparing serum concentrations in the 600 μg treatment group versus the saline treatment group (Supplemental Dataset 1).

**Table 3:** Changes in serum cytokines during immunization

Time	Cytokine^a^	Treatment						
		Adjuvant	10 μg	30 μg	100 μg	300 μg	900 μg	Saline
Week 0	IFN-γ	0	0	0.087	0	0.075	0	0
Week 3		0	−0.019	0.022	0	0	0	0
Week 6		0	0	0.064	0	0	0	0
Week 24		0	0	−0.094	0	0	0	0
Week 0	IL-10	0	0	0.019	0	0	0	0
Week 3		0	0	−0.015	0	0	−0.129	0
Week 6		0	0	0.056	0	0	0	0
Week 24		0	−0.119	−0.072	−0.114	0.166	0	−0.135
Week 0	IL-13	0	0	0.037	0	0	0	0
Week 3		0	0	0.034	0	0	−0.103	0
Week 6		0	0	0.053	0	0	0	0
Week 24		0	0	−0.084	0	0	0	0
Week 0	IL-1β	0	0	0.028	0	0	0	0
Week 3		0	−0.186	−0.002	0	−0.348	−0.287	0
Week 6		0	0.139	0.052	0	0	0	0
Week 24		0	−0.185	−0.108	−0.211	0	−0.104	−0.197
Week 0	IL-2	0	0	−0.077	0	0	0	0
Week 3		0	0	−0.204	0	0	0	0
Week 6		0	0	−0.241	0	0	0	0
Week 24		0	0	−0.306	0	0	0	0
Week 0	IL-4	0	0	0.009	0	0	0	0
Week 3		0	0	0.034	0	0	0	0
Week 6		0	0	0.059	0	0	0	0
Week 24		0	0	−0.473	0	0	0	0
Week 0	IL-6	0.041	0.128	0.318	0.189	0.463	0.986	0.035
Week 3		0.814	0.789	0.329	0.145	0.306	0.441	0
Week 6		0.163	0.592	0.434	0.609	0.332	0.568	0
Week 24		0	0	−0.122	0	0	0	0.158
Week 0	IL-8	−0.162	−0.311	0.068	0.216	−0.005	−0.064	0.134
Week 3		−0.312	−0.372	0.039	−0.255	−0.376	−0.567	−0.121
Week 6		−0.212	−0.439	−0.062	−0.457	−0.076	−0.522	−0.163
Week 24		0.063	−0.365	0.011	−0.189	0.019	0.016	−0.303
Week 0	TNF-α	0.111	0	0.024	0	0	0	0
Week 3		0	0	−0.032	0	0	0	0
Week 6		0	0	0.047	0	0	0	0
Week 24		0	0	−0.118	0	0	0	0

a Data are expressed as log_2_ fold change from predose levels.

## Discussion

Here, we describe the results from preclinical studies in NHPs with a novel anti-PCSK9 vaccine, VXX-401, which consists of a synthetic peptide designed to target PCSK9 and formulated in an alum-based adjuvant (Fig. 1A). Immunization with VXX-401 induces the production of antibodies that recognize and bind to endogenous PCSK9, thus prompting a reduction in LDL-C (Fig. 1B). More specifically, our pharmacology studies in cynomolgus monkeys indicate that VXX-401 is highly immunogenic and reduces LDL-C by 30–40% without alterations in serum HDL-C. In vitro analyses further confirmed that VXX-401 induces the production of anti-PCSK9 antibodies with potent binding properties and blocking properties. In addition, VXX-401 induces a safe humoral response and is well tolerated, with no evidence of autoimmunity or chronic inflammation. Taken together, these findings provide a compelling testament to the potential of VXX-401 as a PCSK9 inhibitor for the treatment of hypercholesterolemia.

PCSK9 vaccines have long been the subject of preclinical efforts, premised on the induction of protective antibodies to neutralize PCSK9-mediated degradation of hepatic LDLR. However, such vaccines face numerous hurdles, beginning with the difficulty to safely overcome immune tolerance. Traditionally, vaccines for endogenous proteins have used mimetic or homolog epitopes with inherent immunogenicity to induce a crossreactive antibody response against of the target of interest. Examples of this include bacterial and viral proteins with structural similarity to self-antigens as well as the derivation of immunostimulatory epitopes from endogenous sequences via mutation-prone polymerases (27). VXX-401, by contrast, relies on the T-helper peptide to drive a humoral response against the otherwise immunosilent epitope of native, human, PCSK9. Antibody responses against PCSK9 may be blunted because of B-cell tolerance toward self-antigens, and in instances where robust titers are successfully induced, it is necessary to test for cellular autoimmunity against PCSK9. Indeed, immunization must induce high-affinity anti-PCSK9 antibodies without triggering a T-cell-specific response against the endogenous protein, which risks lymphocyte infiltration into the liver, chronic inflammation, and the destruction of PCSK9-expressing cells (28). However, follicular T-cells are also crucial to B-cell activation, maturation, proliferation, and differentiation into antibody-secreting plasma cells (29). Accordingly, a PCSK9 vaccine must evoke a highly specific, humoral, immune response without provoking a cellular immune response. This implies that antibody production is only stimulated by re-exposure to the peptide immunogen following the initial prime, and antibody levels wane in the absence of further vaccination. Second is the nature of the target itself: PCSK9 circulates in the blood stream on the order of 100 s of ng per ml (30), which sets a high bar for the requisite antibody quantity to achieve cholesterol-lowering effects. There is also a positive hepatic feedback loop to contend with, wherein PCSK9 inhibitor therapy can enhance the concentration of circulating target protein up to 10-fold above baseline (31). Finally, it is furthermore desirable to lower LDL-C without impacting HDL-C, as a higher HDL-to-LDL ratio is associated with reduced risk of coronary atherosclerotic heart disease (32).

The first PCSK9 vaccines described in 2012 featured a DNA (i.e., plasmid expression vector) and protein-based approach to immunization in dyslipidemic mice. Both treatments induced the production of anti-PCSK9 antibodies, and the protein-based vaccine reduced LDL-C up to 60%. This was accompanied by a 42% reduction in HDL-C, which is directly regulated by PCSK9 in mice (33). A monovalent virus-like particle delivery system leveraged for PCSK9 vaccination was found to be immunogenic in macaques but failed to lower LDL-C unless coupled with simvastatin treatment, whereas a bivalent virus-like particle vaccine was able to achieve a modest 28% reduction in LDL-C (34, 35). Another peptide-based PCSK9 vaccine was tested in *ApoE*-deficient mice but had no discernible impact on LDL-C despite a significant positive correlation between anti-PCSK9 titers and hepatic LDLR surface expression. More problematically, antibody isotyping revealed the presence of IgG2B and C subtypes, suggesting significant potential for a proinflammatory autoimmune response (36). An anti-PCSK9 peptide-based vaccine demonstrated efficacy in *Ldlr*^+/−^ mice but failed to reduce LDL-C in wild-type mice or Wistar rats (37). Finally, the anti-PCSK9 peptide vaccines AT04A and AT06A from AffiRiS AG exhibited robust titers against the peptide epitope but not PCSK9 in a phase I clinical trial and failed to reduce mean LDL-C more than 11.2% and 13.3% from baseline, respectively (38). Demonstrably, there are many challenges associated with developing a safe, well-tolerated, and highly immunogenic vaccine against PCSK9 that yields targeted reductions in LDL-C.

In contrast to other vaccines, immunization of cynomolgus monkeys with VXX-401 induced a safe immune response and robust titers that reached peak levels within a few weeks following a three-dose priming regimen. In the longitudinal studies, antibody titers progressively decreased and returned to baseline within weeks or months following the priming regimen. However, a single administration of a VXX-401 boost was sufficient to restore maximal antibody titers. That the antibody titers progressively decrease after a priming or boost regimen is reassuring from a safety standpoint and suggests that boosting every 2 or 3 months might be necessary to maintain a sustained response. VXX-401 therefore could be used as an alternative to statins, which must be taken every day because of their half-life of several hours, and to mAbs, which have relatively short half-lives (10–20 days), require biweekly subcutaneous injections, and are expensive (39).

Importantly, VXX-401 consistently reduced LDL-C 30–40% in cynomolgus monkeys, which is on part with statin therapy (40). Treatment did not alter serum HDL-C in any of the cohorts examined (supplemental Fig. S2), which is clinically relevant as both lower levels of LDL-C and higher levels of HDL-C are associated with superior resistance to heart attack and stroke (41). Noteworthily, in the pilot study, reductions of serum LDL-C measured over a 2 week period were comparable between the group treated with VXX-401 and the group receiving a bolus injection of evolocumab, a reference mAb against PCSK9, though the mAb produced a greater reduction in LDL-C in the week immediately after dosing. Both the 100 and 600 μg dosage groups from the GLP toxicity study exhibited significantly higher serum levels of total PCSK9 relative to the saline and adjuvant controls by the study end point (23, 42). These findings are consistent with the increase in plasma PCSK9 elicited by monoclonal therapy (i.e., evolocumab), which has been attributed in previous studies to the regulatory loop that governs PCSK9’s secretion (31, 43). Moreover, affinity-purified antibodies from NHP bound to human PCSK9 with a potency comparable to evolocumab and restored the uptake of pHrodo-labeled LDL in HepG2 liver cells even in the presence of PCSK9. These results align with the decrease of serum LDL-C in VXX-401-treated monkeys and demonstrate that high-affinity vaccine-derived antibodies exert a protective effect on the recycling of hepatic LDLR, which translates to increased LDL-C absorption.

With regard to cell-mediated immunity and autoimmunity, VXX-401 was found to safely overcome immune tolerance without generating a targeted T-cell response against PCSK9 or chronic inflammation. As shown by ELISpot, stimulation of PBMCs with the B-cell epitope alone (i.e., p5494a) failed to induce the production of both IFN-γ and IL-4, whereas the T-helper elicited a robust cytokine. Thus, the immune response will naturally wane in the absence of additional boosts, which is consistent with the progressive return to baseline of serum antibody titers and LDL-C in monkeys. Further bolstering the safety profile of VXX-401, analyses of serum samples collected preinjection and postinjection revealed negligible fluctuations in proinflammatory cytokines (Table 3). Overall, the levels of cytokines remained comparable before and after dosing, with no significant differences between any of the groups. These findings were further corroborated by the results of the GLP toxicity study, which did not reveal any significant, sustained, oscillations in IFN-γ, IL-1β, IL-6, or TNF-α. This is noteworthy in so far that IFN-γ and TNF-α are associated with cell-mediated immunity (44), whereas IL-1β and IL-6 are potent proinflammatory cytokines (45, 46). Thus, these results demonstrate that treatment with VXX-401 does not induce a significant proinflammatory response. In addition, VXX-401 specifically induces anti-PCSK9 IgG1. The lack of proinflammatory and allergen-centric subtypes demonstrates the nature of the safe humoral response induced by VXX-401. This is consistent with the clinical data available from UB-311 and UB-312, vaccines against Aβ and α-synuclein using the same peptide vaccine technology as VXX-401, which highlight the platform’s ability to safely break immune tolerance and target endogenous proteins without T-cell-mediated cytotoxicity (19, 20, 47).

In agreement, clinical observations and safety pharmacology assessments made during the treatment and recovery phases of the GLP toxicity study with VXX-401 failed to identify any treatment-related morbidities. Indeed, VXX-401 was found to be safe and well tolerated, with the main side effects being mild injection site reactions and a transient increase in total protein and globulin. Total protein and globulin levels in NHP returned to baseline by week 17, alluding to a stabilization of the immune response in the early weeks of the vaccination regimen. Injection site reactions were also observed in the adjuvant control group and are well-known side effects of alum-containing vaccines, which have demonstrated an excellent safety profile over their 70 year use (48).

Taken together, these results indicate that VXX-401 is a safe and highly effective anti-PCSK9 vaccine that mediates up to a 40% reduction in serum LDL-C via the production of antibodies that inhibit target-mediated LDLR degradation. Cynomolgus monkeys have long been touted as the highest fidelity preclinical model to humans for investigational new drug-enabling studies related to adsorption, distribution, immunogenicity, metabolism, efficacy, and toxicity (49). Consequently, the data presented herein build a compelling case for VXX-401 as a treatment for hypercholesterolemia and the primary prevention of ASCVD. In the event that these findings translate to clinically meaningful (i.e., 25–40%) LDL-C reductions in humans, VXX-401 could be used in combination with other lipid-lowering therapies, as well as a stand-alone treatment for patients with more moderate hypercholesterolemia, or even as a first-line prophylactic therapy in those at risk for ASCVD but who have normal cholesterol levels. As a peptide vaccine, VXX-401 possesses many practical advantages over other cholesterol-lowering medications, including superior cost effectiveness and broader patient accessibility than mAbs and inclisiran (siRNA), as well as less adverse side effects and dosing frequency, which supports lower discontinuation rates and improved patient compliance over statins.

## Data availability

The datasets described herein are available upon request from the corresponding author.

## Supplemental data

This study contains supplemental data.

## Conflict of interest

All studies described herein were funded by Vaxxinity, Inc All authors are past or current Vaxxinity employees and hold company stock/stock options.
