# Supplementary material for: VXX-401, a novel anti-PCSK9 vaccine, reduces LDL-C in cynomolgus monkeys
Source: J Lipid Res. 2024 Jan 10;65(2):100497. doi: 10.1016/j.jlr.2024.100497 (PMC10875594; doi:10.1016/j.jlr.2024.100497)
Supplement: Supplemental data [file mmc1.pdf]

# VXX-401, a novel anti-PCSK9 vaccine, reduces LDL-C in cynomolgus monkeys

## SUPPLEMENTAL MATERIALS

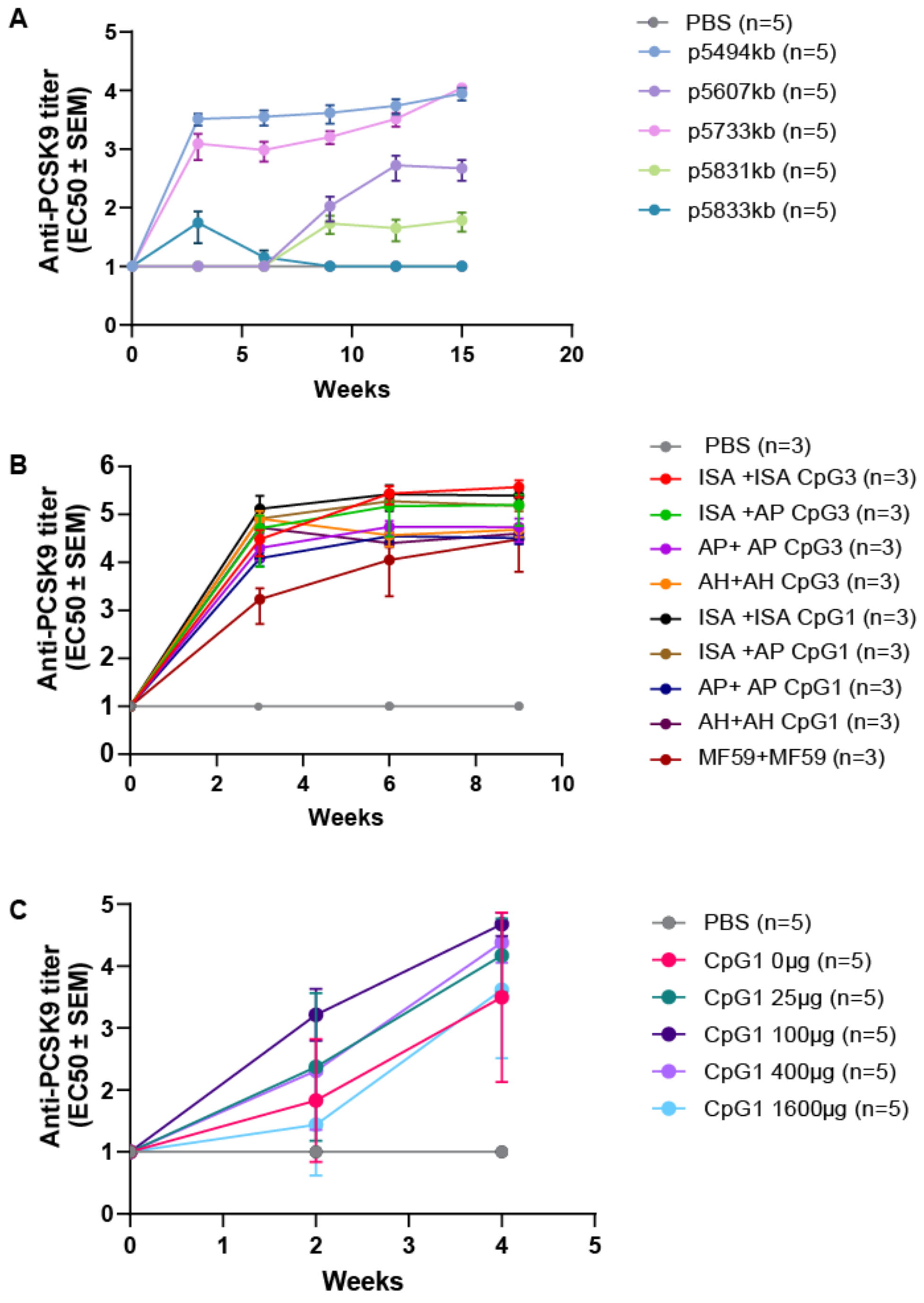


**Supplemental Figure 1. Peptide and adjuvant selection by immunogenicity screening in guinea pigs.** A: Screening of lead PCSK9 B-cell epitope peptides in guinea pigs. B: Adjuvant and excipient testing in guinea pigs for p5494kb. C: CpG1 dose selection study with p5494kb in guinea pigs. Titers are expressed as the $\log_{10}$ value of the $EC_{50}$, with error bars representing the standard error from the mean.

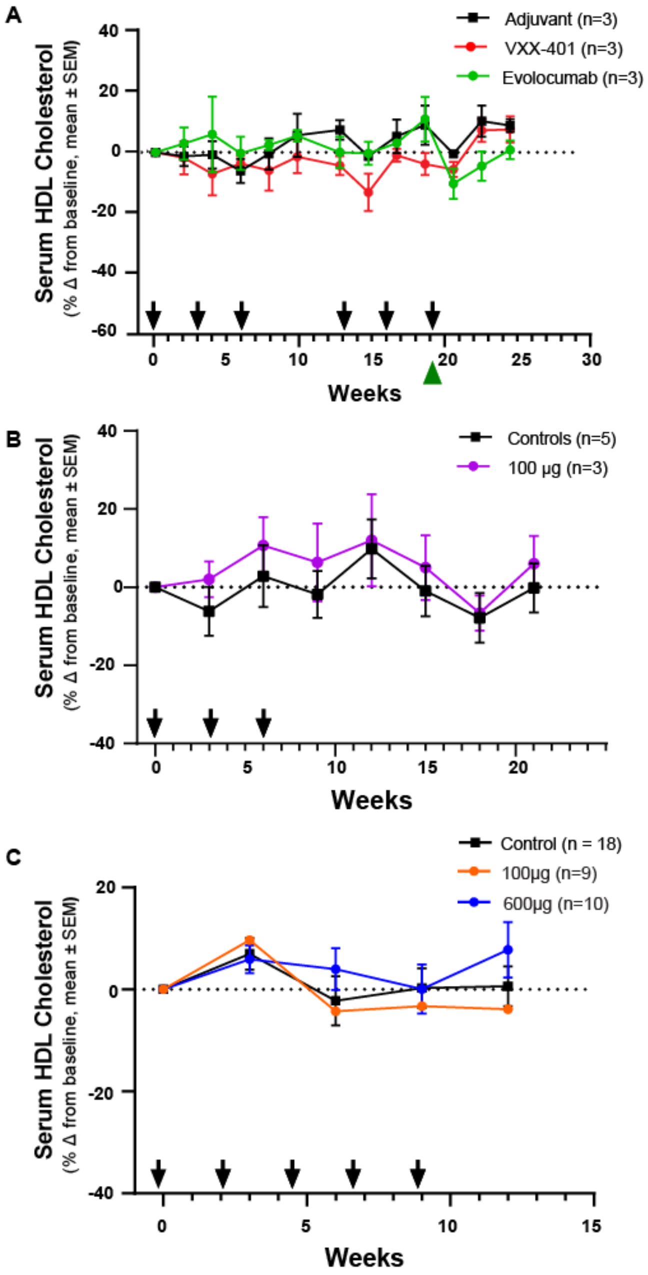


**Supplemental Figure 2. VXX-401 and p5494kb do not cause changes in HDL cholesterol.** A: Two-week HDL-C metrics from the pilot study. The green triangle marks the time at which a comparator group received evolocumab. B: Three-week HDL-C metrics from the dose-ranging study; data shown are for the priming regimen. C: Three-week HDL-C metrics from the GLP toxicity study. Arrows indicate prime and boost injections. All titers are expressed as the $log_{10}$ value of the $EC_{50}$. HDL-C is shown as percent change from baseline. Error bars represent the standard error of the mean.

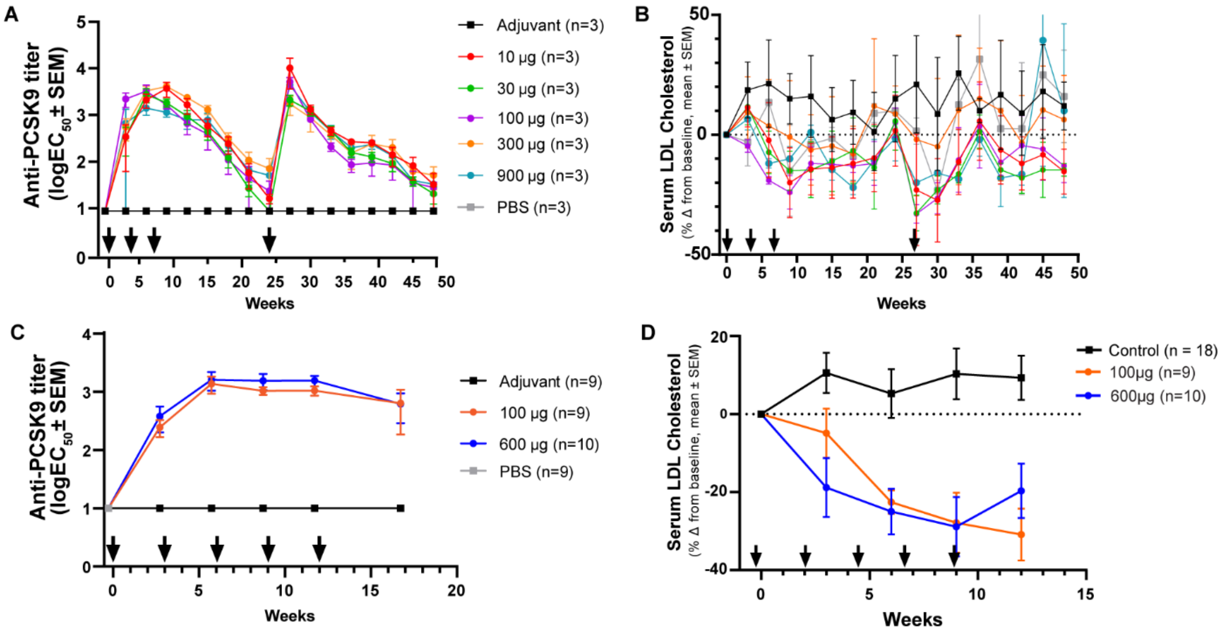


**Supplemental Figure 3. Vaccination against p5494a elicits a robust antibody response in cynomolgus monkeys and significantly reduces serum levels of LDL-C.** A and B: Anti-PCSK9 titers and three-week LDL-C metrics from the dose-ranging study for all dosage and control groups. The green triangle marks the time at which a comparator group received evolocumab. C and D: Anti-PCSK9 titers and three-week LDL-C metrics from the GLP toxicity study for all dosage and control groups. Arrows indicate the timeline of the prime and boost schedule. All titers are expressed as the $\log_{10}$ value of the $EC_{50}$. LDL-C is presented as percent change from baseline. Error bars represent the standard error of the mean.

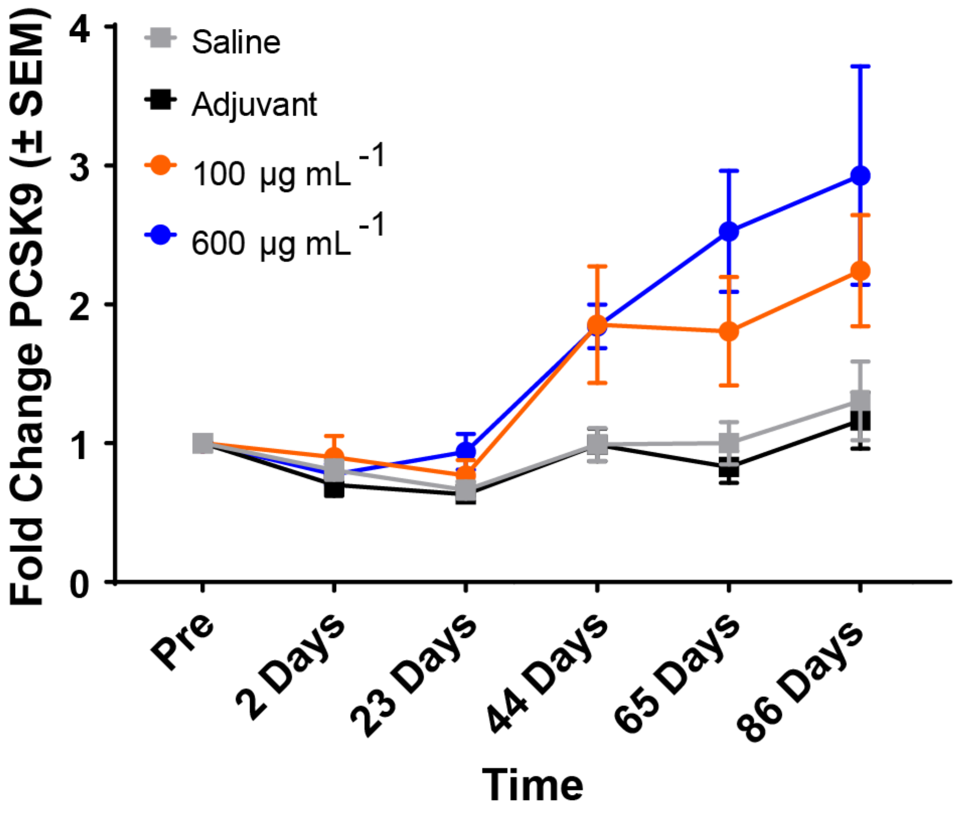


**Supplemental Figure 4. VXX-401 immunization increases total PCSK9 in the serum of non-human primates.** Total PCSK9 was quantified in cynomolgus monkey serum from the saline and adjuvant controls, as well as the 100 and 600 µg dosage groups of the GLP toxicity study. Fluctuations from baseline are expressed as fold change from pre-bleed. Error bars represent the standard error of the mean.

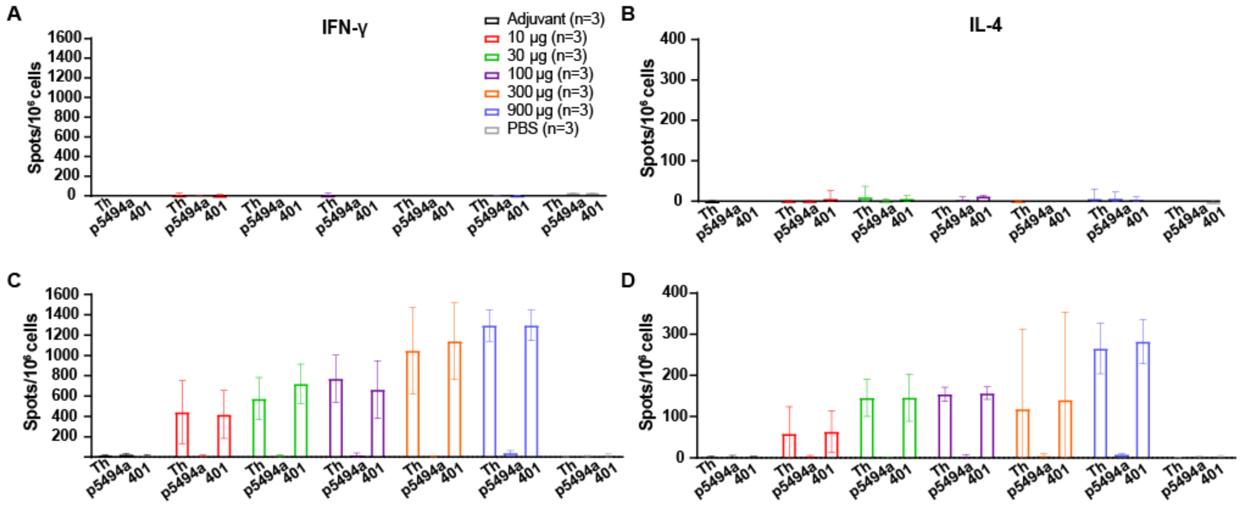


**Supplemental Figure 5. VXX-401 does not induce autoimmunity against endogenous PCSK9.** A and B: Spot forming units (SFU) per million PBMC for IFN-γ and IL-4 pre-immunization with VXX-401 for all dosage and control groups from the dose-ranging study. C and D: SFU per million PBMC for IFN-γ and IL-4, post-immunization with VXX-401 for all dosage and control groups from the dose-ranging study. Bar chart values indicate the group average. Error bars represent the standard error of the mean.

## SUPPLEMENTAL METHODS

### Measurement of total PCSK9 by sandwich ELISA assay

To quantify total serum PCSK9 in samples from the GLP toxicity study, high-bind ELISA 96 well plates were coated with rat, anti-human, PCSK9 antibody that was affinity purified from the hyperimmune serum of rats immunized with VXX-401. Plates were coated overnight at 2-8°C with antibody at a final concentration of 2 µg/mL in 1X PBS. The next day, assay plates were twice blocked with PBS-based Super Block Blocking Buffer for 1 h at 30°C with shaking at 500 rpm. For standards, human recombinant PCSK9 was serially diluted 2-fold over an 8-point curve from a starting concentration of 1,500 ng/mL. Serum samples were diluted 1:20 and 1:50 in 2% BSA and 1X PBS then transferred to assay plates and incubated for 1 h at 25°C without shaking. Next, evolocumab was added to the assay plates at a final concentration of 10 µg/mL and incubated as in the previous step. Afterwards, donkey anti-human IgG HRP was diluted 1:20,000 in 5% BSA in 1X PBS and added to each plate, followed by incubation at 25°C without shaking for 1 h. TMB Ultra ELISA substrate was subsequently added to the plates and allowed to develop for 15 minutes protected from light, after which 2 N sulfuric acid was added to halt the reaction. The plates were read at an absorbance of 450 nm within 10 minutes of halting the reaction, after which total PCSK9 was back calculated from the standard curve for within-range samples.

**SUPPLEMENTAL DATASET 1**

**Serum cytokine profiles for the VXX-401 GLP toxicity study**

| | | | baseline | post day 1 dose | post day 22 | Post day 43 dose | post day 64 | post-day 85 |
|---|---|---|---|---|---|---|---|---|
| **Group (Dose; µg)** | **Animal ID** | **Sex** | **IL-1β (pg/mL)** | **IL-1β (pg/mL)** | **IL-1β (pg/mL)** | **IL-1β (pg/mL)** | **IL-1β (pg/mL)** | **IL-1β (pg/mL)** |
| 1 (0; Saline Control) | A369 | M | | 1.36 | NA | 3.18 | 1.83 | 1.28 |
| | A381 | M | 5.77 | 7.13 | 23.07 | 14.98 | 11.62ǂ | 15.51 |
| | A387 | M | 0.79 | | 1.13 | 1.33 | 0.67 | BLOQ |
| | A388R | M | | | 3.45 | 4.72 | 1.46 | 1.48 |
| | A397R | M | 2.06 | 1.19 | 2.36 | 2.57 | 1.15 | 0.9 |
| | A529 | F | 13.65 | 9.5 | BLOQ | 2.41 | 22.95 | 10.67 |
| | A538 | F | | | BLOQ | BLOQ | BLOQ | BLOQ |
| | A561 | F | | 1.08 | BLOQ | 0.81 | 2.35 | 1.61 |
| | A570R | F | 9.37 | 7.89 | 8.97 | 12.29 | 8.66 | 9.63 |
| 2 (0; Adjuvant | B393 | M | 5.95 | 2.63 | 7.5 | 1.33 | 1.2 | 0.48 |
| | B394 | M | 0.85 | BLOQ | BLOQ | 5.7 | BLOQ | 1.83 |
| | B395 | M | BLOQ | 1.29 | BLOQ | BLOQ | BLOQ | 1.15 |
| | B400R | M | 0.86 | BLOQ | 1.33 | 1.13 | 3.47 | 0.86 |
| | B530 | F | 0.79 | BLOQ | 2.18 | 0.74 | 1 | BLOQ |
| | B533 | F | 0.63 | BLOQ | 1.59 | BLOQ | 1.08 | 0.22 |
| | B552 | F | BLOQ | 1.39 | 1.59 | BLOQ | BLOQ | 1.26 |
| | B557R | F | 0.97 | BLOQ | 4.71 | 3.87 | 2.76 | 2.24 |
| | B566R | F | 1.6 | 1.03 | 1.25 | BLOQ | 0.9 | BLOQ |
| 3 100 µg | C382 | M | 5.02 | 7.12 | 15.66 | 8.98 | BLOQ | 3.81 |
| | C389 | M | 0.48 | 1.2 | BLOQ | BLOQ | 1.6 | 1.15 |
| | C401 | M | 1.6 | BLOQ | 1.76 | 1.89 | BLOQ | BLOQ |
| | C402R | M | BLOQ | 1.39 | 1.16 | BLOQ | 2.12 | BLOQ |
| | C409R | M | 7.04 | 1.48 | 4 | 2.35 | 4.16 | 1.26 |
| | C518 | F | 0.68 | BLOQ | BLOQ | BLOQ | 0.95 | BLOQ |
| | C541 | F | 0.36 | 1.48 | BLOQ | 1.01 | 1.15 | BLOQ |
| | C549 | F | 0.9 | BLOQ | BLOQ | 1.15 | BLOQ | BLOQ |
| | C592R | F | 3.14 | 3.49 | 2.57 | 3.1 | 1.2 | BLOQ |
| 4 600 µg | D384 | M | 0.85 | BLOQ | 6.92 | 1.18 | BLOQ | BLOQ |
| | D385 | M | 0.48 | 1.25 | BLOQ | 0.67 | 9.06 | BLOQ |
| | D386 | M | BLOQ | BLOQ | 1.16 | 4.2 | 5.56 | 4.2 |
| | D396R | M | BLOQ | 1.2 | 4.86 | 1.52 | 1 | BLOQ |
| | D407R | M | 0.85 | 1.39 | 5.35 | 0.81 | 7.53 | BLOQ |
| | D523 | F | 0.97 | BLOQ | 4.43 | 6.54 | 3.29 | BLOQ |
| | D545 | F | 0.63 | BLOQ | BLOQ | BLOQ | 0.77 | BLOQ |
| | D548 | F | BLOQ | BLOQ | 1.07 | 1.09 | BLOQ | 3.59 |
| | D577R | F | 0.57 | 1.11 | 1.16 | 2.3 | 1.34 | 2.66 |
| | D583R | F | BLOQ | 1.34 | 2.09 | 1.2 | BLOQ | 0.78 |

IL-1β serum cytokine profiles from the GLP toxicity study. BLOQ = below limit of quantification.

| Group (Dose; µg) | Animal ID | Sex | baseline<br>IL-6 (pg/mL) | post day 1 dose<br>IL-6 (pg/mL) | post day 22<br>IL-6 (pg/mL) | Post day 43 dose<br>IL-6 (pg/mL) | post day 64<br>IL-6 (pg/mL) | post-day 85<br>IL-6 (pg/mL) |
|---|---|---|---|---|---|---|---|---|
| 1 | A369 | M | 9.73 | 6.31 | NA | 6.6 | 7.82 | 7.00ǂ |
| (0; Saline Control) | A381 | M | 39.56 | 51.08 | 185.07ǂ | 84.48 | 113.87 | 103.93 |
| | A387 | M | BLOQ | BLOQ | BLOQ | 6.21 | 5.31 | 12.57 |
| | A388R | M | 13.15 | 18.88 | 30.06 | 16.34 | 11.34 | 13.69 |
| | A397R | M | 20.44 | 19.23 | 40.44 | 13.12 | 19.01 | BLOQ |
| | A529 | F | 57.79 | 74.34 | BLOQ | 43.17 | 99.79 | 56.57 |
| | A538 | F | BLOQ | 25.09 | BLOQ | 8.69 | 5.31 | BLOQ |
| | A561 | F | 14.49 | BLOQ | BLOQ | 8.69 | 7.98 | 11.29 |
| | A570R | F | 60.11 | 80.05 | 60.46 | 135.13 | 116.06 | 79.4 |
| 2 | B393 | M | 20.44 | 15.74 | 14.19 | 17.67 | 13.43 | BLOQ ǂ |
| (0; Adjuvant Control) | B394 | M | 9.73 | 25.96 | 9.87 | 20.68 | 14.71 | 35.5 |
| | B395 | M | BLOQ | 22.16 | BLOQ | 13.91 | 10.85 | 21.43 |
| | B400R | M | BLOQ | BLOQ | 9.61 | 12.62 | 25.76 | 6.56 |
| | B530 | F | BLOQ | BLOQ | 13.05 | 21.18 | 22.08 | 29.71 |
| | B533 | F | BLOQ | 16.86 | 4.78 | 4.11 | 6.89 | BLOQ |
| | B552 | F | 33.99 | 25.09 | 22.88 | 17.76 | 16.84 | 28.12 |
| | B557R | F | 8.2 | 14.5 | 14.7 | 12.74 | 61.28 | 45.97 |
| | B566R | F | 25.49 | 28.48 | 13.12 | 7.98 | 14.71 | 8.97 |
| 3 | C382 | M | 35.8 | 80.59 | 87.94 | 102.96 | 121.91 | 32.46 |
| 100 µg | C389 | M | BLOQ | BLOQ | 28.56 | 38.08 | 55.04 | 29.69 |
| | C401 | M | 8.2 | 19.23 | 8.03 | 23.96 | 64.36 | 32.6 |
| | C402R | M | BLOQ | BLOQ | 14.7 | 74.99 | 41.65 | 18.27 |
| | C409R | M | 75.58 | 47.31 | 29.72 | 33.73 | 128.05 | 35.5 |
| | C518 | F | 29.04 | 23.87 | 14.27 | 19.23 | 14.71 | 13.65 |
| | C541 | F | 23.85 | 20.67ǂ | 8.03 | 18.04 | 39 | 34.95 |
| | C549 | F | BLOQ | BLOQ | 6.21 | 8.96 | 6.56 | 4.14 |
| | C592R | F | 12.29 | 37.37 | 15.28 | 63.37 | 30.69 | 11.68 |
| 4 | D384 | M | BLOQ | BLOQ | 63.81 | 58.89 | 25.05 | 18.11 |
| 600 µg | D385 | M | BLOQ | 57.45 | 25.21 | 29.01 | 60.21 | 55.71 |
| | D386 | M | BLOQ | 38.66 | 28.97 | 78.66 | 39.54 | 61.38 |
| | D396R | M | BLOQ | BLOQ | 31.33 | 26.55 | 103.01 | 33.11 |
| | D407R | M | 9.73 | 16.86 | 47.06 | 30.66 | 83.56 | 33.4 |
| | D523 | F | 13.15 | BLOQ | 34.33 | 90.1 | 50.84 | 68.16 |
| | D545 | F | BLOQ | 28.21 | 76.41 | 41.57 | 78.86 | 21.43 |
| | D548 | F | BLOQ | 14.5 | 13.12 | 8.69 | 23.56 | 33.56 |
| | D577R | F | 13.15 | 25.88 | 51.81 | 32.59 | 54.79 | 90.42 |
| | D583R | F | BLOQ | 17.9 | 67.18 | 56.87 | 39.54 | 54.89 |

IL-6 serum cytokine profiles from the GLP toxicity study. BLOQ = below limit of quantification.

| | | | baseline | post day 1 dose | post day 22 | Post day 43 dose | post day 64 | post-day 85 |
|---|---|---|---|---|---|---|---|---|
| **Group (Dose; µg)** | **Animal ID** | **Sex** | **IFN-γ (pg/mL)** | **IFN-γ (pg/mL)** | **IFN-γ (pg/mL)** | **IFN-γ (pg/mL)** | **IFN-γ (pg/mL)** | **IFN-γ (pg/mL)** |
| 1 | A369 | M | 7.52 | 11.67 | NA | 17.54 | 16.42 | 7.46 |
| (0; Saline Control) | A381 | M | 38.87 | 58.51 | 71.67 | 99.95 | 82.68 | 109.87 |
| | A387 | M | 4.66 | 3.8 | 8.14 | 6.94 | 6.12 | 9.65 |
| | A388R | M | 19.37 | 8.36 | 12.8 | 21.09 | 7.85 | 10.14 |
| | A397R | M | 8.41 | 3.59 | 3.79 | 5.25 | 2.2 | BLOQ |
| | A529 | F | 46.47 | 51.77 | BLOQ | 48.75 | 77.26 | 93.65 |
| | A538 | F | BLOQ | 0.35 | 2.59 | 2.13 | 2.15 | 2.98 |
| | A561 | F | 2.49 | 0.96 | 1.98 | 4.48 | 7.28 | 7.18 |
| | A570R | F | 10.59 | 10.17 | 11.7 | 19.34 | 14.01 | 15.07 |
| 2 | B393 | M | 11.09 | 7.91 | 8.95 | 1.67 | 1.37 | 1.3 |
| (0; Adjuvant Control) | B394 | M | 8.23 | 4.02 | 5.36 | 10.15 | 3.63 | 8.41 |
| | B395 | M | 4.74 | 1.76 | 4.18 | 4.43 | 5.95 | 16.23 |
| | B400R | M | 3.08 | 4.1 | 7.8 | 6.99 | 33.25 | 4.58 |
| | B530 | F | 7.88 | 7.58 | 14 | 7.57 | 8.91 | 5.86 |
| | B533 | F | 3.42 | 3.82 | 4.35 | 3.22 | 1.86 | 3 |
| | B552 | F | 3.51 | 1.22 | 3.77 | 2.43 | 2.31 | 4.32‡ |
| | B557R | F | 7.74 | 11.1 | 30.51 | 26.21 | 25.5 | 20.14 |
| | B566R | F | 11.72 | 6.42 | 7.39 | 3.12 | 2.08 | 9.01 |
| 3 | C382 | M | 13.2 | 15.75 | 42.27 | 32.21 | 1.78 | 19.12 |
| 100 µg | C389 | M | BLOQ | 0.94 | 2.12 | 1.61 | 14.53 | 6.19 |
| | C401 | M | 11.59 | 6.76 | 7.94 | 7.53 | 7.58 | 26.31 |
| | C402R | M | 2.29 | 0.74 | 11.57 | 10.01 | 77.46 | 9.89 |
| | C409R | M | 163.7 | 105.2 | 65.57 | 61.25 | 20.13 | 77.14 |
| | C518 | F | 14.09 | 4.93 | 6.11 | 5.47 | 4.44 | 4.84 |
| | C541 | F | 14.68 | 12.56 | 11.83 | 9.37 | 19.32 | 9.5 |
| | C549 | F | 5.46 | 5.14 | 7.14 | 5.4 | 1.02 | BLOQ |
| | C592R | F | 14.77 | 18.45 | 11.39 | 25 | 9.37 | 7.85 |
| 4 | D384 | M | 4.16 | 2.72 | 10.07 | 5.33 | 9.21 | 5.62 |
| 600 µg | D385 | M | BLOQ | 0.86 | 9.4 | 9.69 | 46.27 | 19.02 |
| | D386 | M | 8.23 | 5.46 | 8.42 | 49.27 | 53.63 | 23.68 |
| | D396R | M | 4.49 | 3.4 | 40.58 | 9.93 | 9.97 | 13.61 |
| | D407R | M | BLOQ | 0.75 | 9.79 | 1.55 | 20.31 | BLOQ |
| | D523 | F | 15.99 | 16.08 | 20.97 | 35.82 | 21.32 | 20.6 |
| | D545 | F | 2.29 | 4.05 | 4 | 7.16 | 2.14 | 3.26 |
| | D548 | F | 3.45 | 1.59 | 1.46 | 2.32 | 2 | 18.62 |
| | D577R | F | 6.08 | 4.38 | 8.57 | 8.98 | 4.23 | 8.52 |
| | D583R | F | 2.29 | 1.76 | 6.42 | 3.91 | 2.23 | 3.96 |

IFN-γ serum cytokine profiles from the GLP toxicity study. BLOQ = below limit of quantification.

| Group (Dose; µg) | Animal ID | Sex | baseline TNF-α (pg/mL) | post day 1 dose TNF-α (pg/mL) | post day 22 TNF-α (pg/mL) | Post day 43 dose TNF-α (pg/mL) | post day 64 TNF-α (pg/mL) | post-day 85 TNF-α (pg/mL) |
|---|---|---|---|---|---|---|---|---|
| 1 | A369 | M | 7.3 | 13.79 | NA | 43.89 | 7.74 | 9.58 |
| (0; Saline Control) | A381 | M | 29.62 | 55.26 | 170.59 | 213.28 | 208.61 | 133.17 |
| | A387 | M | BLOQ | BLOQ | 3.25 | BLOQ | BLOQ | 7.46 |
| | A388R | M | 6.86 | BLOQ | 9.05 | 29.14 | BLOQ | 5.12 |
| | A397R | M | 14.9 | 6.31 | 9.67 | 23.5 | 5.6 | 7.41 |
| | A529 | F | 69.14 | 66.26 | BLOQ | 50.33 | 85.78 | 44.08 |
| | A538 | F | BLOQ | BLOQ | BLOQ | BLOQ | BLOQ | 4.28 |
| | A561 | F | 5.83 | BLOQ | BLOQ | BLOQ | 8.03 | 8.06 |
| | A570R | F | 33.07 | 26.92 | 78.7 | 76.75 | 49.98ǂ | 64.36 |
| 2 | B393 | M | 19.04 | 10.73 | 43.33 | BLOQ | 5.96 | BLOQ |
| (0; Adjuvant | B394 | M | 7.78 | BLOQ | BLOQ | 19.89 | 4.12 | 10.66 |
| | B395 | M | 8.64 | BLOQ | BLOQ | BLOQ | 10.13 | 37.24 |
| | B400R | M | 6.27 | 4.25 | 5.22 | BLOQ | 14.81 | 3.34 |
| | B530 | F | 9.89 | 4.61 | 30.1 | BLOQ | 8.16 | BLOQ |
| | B533 | F | 5.38 | BLOQ | BLOQ | BLOQ | 4.12 | BLOQ |
| | B552 | F | 8.22 | BLOQ | 18.91 | BLOQ | 4.87 | 5.21 |
| | B557R | F | 8.64 | 4.61 | 32.87 | 18.1 | 30.46 | 20.92 |
| | B566R | F | 18.51 | 6.22 | 22.79 | BLOQ | 3.33 | BLOQ |
| 3 | C382 | M | 22 | 105.11 | 132.43 | 51.66 | 3.28 | 24.36 |
| 100 µg | C389 | M | BLOQ | BLOQ | BLOQ | BLOQ | 11.89 | 3.3 |
| | C401 | M | 15.6 | 9.06 | BLOQ | 10.73 | 6.31 | 18.61 |
| | C402R | M | BLOQ | BLOQ | BLOQ | BLOQ | 41.83 | BLOQ |
| | C409R | M | 176.34 | 70.37 | 142.03 | 65.44 | 26.45 | 42.41 |
| | C518 | F | 8.04 | BLOQ | BLOQ | BLOQ | 4.87 | BLOQ |
| | C541 | F | 9.07 | 10.16 | BLOQ | BLOQ | 6.7 | BLOQ |
| | C549 | F | BLOQ | BLOQ | BLOQ | 2.64 | 4.12 | BLOQ |
| | C592R | F | 6.69 | 14.1 | 22.84 | 16.49 | 8.16 | BLOQ |
| 4 | D384 | M | 5.83 | 4.95 | 15.73 | BLOQ | 6.92 | BLOQ |
| 600 µg | D385 | M | BLOQ | BLOQ | 18.91 | 8.03 | 65.47 | 10.95 |
| | D386 | M | 8.22 | BLOQ | 23.5 | 55.65 | 34.49 | 20.13 |
| | D396R | M | 3.88 | BLOQ | 91.03 | 9.48 | 7.51 | BLOQ |
| | D407R | M | BLOQ | BLOQ | 50.62 | BLOQ | 9.51 | BLOQ |
| | D523 | F | 24.83 | 10.19 | 42.26 | 19.66 | 11.39 | BLOQ |
| | D545 | F | 4.91 | BLOQ | 5.53 | BLOQ | BLOQ | BLOQ |
| | D548 | F | 5.83 | 4.95 | BLOQ | BLOQ | 4.71 | 33.6 |
| | D577R | F | 11.09 | 7.25 | 20.43 | 12.03 | 6.71 | 7.74 |
| | D583R | F | 4.91 | 4.61 | 22.84 | 9.48 | 6.34 | BLOQ |

TFN-α serum cytokine profiles from the GLP toxicity study. BLOQ = below limit of quantification.

## SUPPLEMENTAL DATASET 2

**VXX-401 GLP Toxicity Final Report Summary**

# 12-Week Intramuscular Injection Toxicity Study of Anti-PCSK9 Vaccine in Cynomolgus Monkeys with a 4-Week Recovery Period

## Final Report

## IITRI Project No. 316000100101

**Testing Facility**:

IIT Research Institute (IITRI)
10 West 35th Street
Chicago, IL 60616

**Study Director**:

William D. Johnson, Ph.D., D.A.B.T.

**Sponsor**:

Vaxxinity
1717 Main Street, Suite 3388
Dallas, TX 75201

**Sponsor Representative**:

Jean-Cosme Dodart, Ph.D.

**Sponsor Project Manager**:

Valorie Ryan, Ph.D.

**Study Initiation Date:** May 17, 2022

**Study Completion Date:** February 9, 2023

**12-WEEK INTRAMUSCULAR INJECTION TOXICITY STUDY OF ANTI-PCSK9 VACCINE IN CYNOMOLGUS MONKEYS WITH A 4-WEEK RECOVERY PERIOD**

## FOREWORD

| | |
|---|---|
| Study Initiation Date: | May 17, 2022 |
| Experimental Initiation Date: | May 24, 2022 (males)<br>May 31, 2022 (females) |
| Experimental Termination Date: | September 13, 2022 (males)<br>September 20, 2022 (females) |

This report describes a toxicity and immunogenicity study conducted by IIT Research Institute (IITRI) for Vaxxinity under IITRI project number 316000100101. The Sponsor Representative was Jean-Cosme Dodart, Ph.D. The Sponsor Project Manager was Valorie Ryan, Ph.D. Supervisory IITRI personnel were as follows:

| | |
|---|---|
| Study Director: | William D. Johnson, Ph.D., D.A.B.T. |
| President and Director: | David L. McCormick, Ph.D., D.A.B.T. |
| Immunogenicity and Cytokine Analyses Contributing Scientist: | Anya Nikolai-Yogerst, Ph.D. |
| Electrocardiographic Examination Contributing Scientist: | Jeffrey Richig, D.V.M. |
| Veterinarian: | Kelly Garcia, D.V.M., Ph.D., D.A.C.L.A.M. |
| Quality Assurance Unit Manager: | Sibby Cantrell Gordon, M.S., R.Q.A.P.-G.L.P. |

Additional supervisory personnel were as follows:

| | |
|---|---|
| Toxicologic Pathology: | Maureen T. O'Brien, D.V.M., M.S., D.A.C.V.P.<br>Charles River Laboratories, Inc.<br>8025 Lamon Ave, Suite 447<br>Skokie, IL 60077<br>15 Worman's Mill Ct., Suite I<br>Frederick, MD 21701 |
| Serum PCSK9 Analysis: | Madeline M. Vroom, Ph.D. and<br>Matthew S. Longo<br>Vaxxinity Discovery Research and Development Laboratory<br>505 Odyssey Way<br>Merritt Island, FL 32953 |

Report Approval:

DocuSigned by:
William D. Johnson
Signer Name: William D. Johnson
Signing Reason: I approve this document
Signing Time: 2023-02-09 | 19:55 CST
B5CD9B71A677444790FAFDC7FE421027

William D. Johnson, Ph.D., D.A.B.T.
Study Director

DocuSigned by:
David L. McCormick
Signer Name: David L. McCormick
Signing Reason: I approve this document
Signing Time: 2023-02-10 | 09:09 PST
3D9A7742ED6A4E408713C397103C20C8

David L. McCormick, Ph.D., D.A.B.T.
President and Director

## GLP COMPLIANCE STATEMENT

This study was conducted in compliance with the U.S. Food and Drug Administration (FDA) Good Laboratory Practice (GLP) Regulations (*Code of Federal Regulations* Title 21 Part 58) and – per the mutual acceptance of data (MAD) agreement – in accordance with the Organization for Economic Cooperation and Development (OECD) Principles of Good Laboratory Practice [OECD ENV/MC/CHEM(98)17], with the exceptions that a retention sample of the saline control was not retained, and the immunogenicity analyses were not conducted in compliance with GLP regulations. The study raw data have been reviewed, and the information contained in this report is an accurate representation of the data within the context of the study design and evaluation criteria.

DocuSigned by:

William D. Johnson

Signer Name: William D. Johnson
Signing Reason: I approve this document
Signing Time: 2023-02-09 | 16:39 CST
B5CD9B71A677444790FAFDC7FE421027

William D. Johnson, Ph.D., D.A.B.T.
Study Director

## QUALITY ASSURANCE STATEMENT

Study Title: 12-Week Intramuscular Injection Toxicity Study of Anti-PCSK9 Vaccine in Cynomolgus Monkeys with a 4-Week Recovery Period

Project Number: 316000100101

Study Director: William D. Johnson, Ph.D., D.A.B.T.

This study has been inspected and the report [with the exceptions of Appendix F (Immunogenicity Analysis Report), Appendix G (Cytokine Analysis Report) and Appendix H (Serum PCSK9 Analysis Report), which were not conducted in compliance with GLP regulations, and Appendix I (Pathology Report), which was audited by the Test Site's Quality Assurance Unit] has been audited by the IITRI Quality Assurance Unit in accordance with U.S. Food and Drug Administration (FDA) Good Laboratory Practice Regulations as set forth in the *Code of Federal Regulations,* Title 21 Section 58.35 and – per the mutual acceptance of data (MAD) agreement – in accordance with the Organization for Economic Cooperation and Development (OECD) Principles of Good Laboratory Practice. The report describes the methods and procedures used in the study and the reported results accurately reflect the raw data of the study. The following are the inspection dates, phases inspected, and the dates the inspection findings were reported:

| | | Findings Reported to: | |
|---|---|---|---|
| Date of Inspection | Phase | Study Director | Management |
| 5/3/2022 | Review protocol | 5/3/2022 | 5/3/2022 |
| 5/24/2022 | Inspect test/control article dispensation, physical exams, body weights/temperatures, site reactogenicity, dosing | 5/24/2022 | 5/24/2022 |
| 8/5/2022 | Review protocol amendment no. 1 | 8/5/2022 | 8/5/2022 |
| 8/19/2022 | Inspect Day 88 blood collection, necropsy and organ weights | 8/19/2022 | 8/19/2022 |
| 9/2/2022 | Review protocol amendment no. 2 | 9/2/2022 | 9/2/2022 |
| 9/12/2022 | Review protocol amendment no. 3 | 9/12/2022 | 9/12/2022 |
| 10/28/2022 | Review protocol amendment nos. 4 and 5 | 10/28/2022 | 10/28/2022 |
| 12/20,22-23,27-30/2022; 1/3-6,9/2023 | Audit main data, main draft report (excluding the appendices) | 1/10/2023 | 1/10/2023 |
| 12/27-28,30/2022 | Audit clinical pathology data and draft report | 12/30/2022 | 12/30/2022 |
| 12/30/2022; 1/3-4/2023 | Audit draft report summary tables, Appendices C and D, and ECG data | 1/5/2023 | 1/5/2023 |
| 1/18,23,25-27,30-31/2023 | Review protocol deviation no.1; check corrections to data and draft report | 1/31/2023 | 1/31/2023 |
| 2/2/2023 | Review protocol deviation no. 2 | 2/2/2023 | 2/2/2023 |
| 2/7/2023 | Audit final report | 2/7/2023 | 2/7/2023 |

## QUALITY ASSURANCE STATEMENT

DocuSigned by:

Sibby Cantrell Gordon

Signer Name: Sibby Cantrell Gordon
Signing Reason: I have reviewed this document
Signing Time: 2023-02-09 | 16:31 CST

E4ECB64913A54E4BAA9BD440D65097C9

Sibby Cantrell Gordon, M.S., R.Q.A.P.-G.L.P.
Manager, Quality Assurance Unit

## SUMMARY

The objective of the study was to determine the toxicity following five intramuscular injections over 12 weeks (on Days 1, 22, 43, 64 and 85) with anti-PCSK9 vaccine in cynomolgus monkeys, with a 4-week recovery period. The study design was as follows:

| Group | Treatment | Peptide Dose (µg) | Number of Monkeys | | | |
|---|---|---|---|---|---|---|
| | | | Main Study | | Recovery | |
| | | | M | F | M | F |
| 1 | Saline Control | 0 | 3 | 3 | 2 | 1 |
| 2 | Adjuvant Control | 0 | 3 | 3 | 1 | 2 |
| 3 | Vaccine | 100 | 3 | 3 | 2 | 1 |
| 4 | Vaccine | 600 | 3 | 3 | 2 | 2 |

Experimental endpoints consisted of moribundity/mortality and clinical observations; physical examination observations; injection site reactogenicity (erythema and edema); body weight; food consumption; body temperature; cardiovascular evaluations [electrocardiography (ECG) tracings, heart rate and rhythm, blood pressure, duration of the PR interval, QRS complex and QT interval]; respiratory function evaluations (respiratory rate and hemoglobin oxygen concentration); central nervous system evaluation (motor activity, behavioral changes, coordination and sensory/motor reflex responses); clinical pathology parameters (clinical chemistry, hematology, coagulation and urinalysis); serum immunogenicity analysis; gross pathology; and histopathological evaluations.

Intramuscular administration of anti-PCSK9 on Days 1, 22, 43, 64 and 85 at dose levels of 100 and 600 µg to cynomolgus monkeys did not result in any test article-related mortality or clinical signs of toxicity. For reactogenicity, in general, erythema was observed on the day of or the day after dosing and was no longer apparent at 48 hours post-dose. There were no treatment-related effects on body weight, body weight gain, food consumption or body temperature. No treatment-related effects on the central nervous system were seen in any monkey. No treatment-related effects on blood pressure, electrocardiographic or respiratory parameters were seen in any monkey. No treatment-related changes were seen in any clinical pathology parameters. Low density lipoprotein levels were decreased, although not statistically significantly, in males at both dose levels on Days 23 (high dose only), 44, 65 and 86, and in females at both dose levels on Day 86, thus demonstrating the intended pharmacological effect of the anti-PCSK9 vaccine. No treatment-related effects on absolute or relative organ weights were seen at the end of the 12-week treatment period.

Anti-PCSK9 titers were not detectable at any time point in animals administered saline or adjuvant only. Titers were detectable by Day 23 (i.e., following the second dose) in animals

## SUMMARY

administered 100 or 600 µg of anti-PCSK9. When comparing geometric group mean titers, female samples demonstrated a dose-dependent increase in titers at Days 44, 65, and 86. However, females that were administered 600 µg/dose of anti-PCSK9 had a reduced titer at the end of the recovery period (i.e., at Day 113), whereas the one surviving female that was administered 100 µg/dose had an increased titer. Since data is only available for one female at this time point, it is not possible to determine whether this was significant. Results for males administered 100 or 600 µg/dose had high standard deviations; therefore, no discernable trend was seen. Samples from males did not show the same reduction in titers at the end of the recovery period that were seen in the females administered 600 µg/dose.

No significant changes were observed in analyzed cytokine levels in test or adjuvant control article-treated study monkeys in comparison to Group 1 (Saline Control).

Overall, female NHP were observed to possess slightly higher PCSK9 levels in their experimental sera than their male counterparts. However, normalizing the data to the pre-bleed values revealed similar fold-change values in the control and vaccine treatment groups. In both the high and low dose treatment groups, serum PCSK9 was observed to increase over time. The vaccine treatment groups performed comparably to one another but yielded greater increases in serum PCSK9 relative to both controls, with the saline and adjuvant groups exhibiting stable serum levels of PCSK9 at all timepoints examined.

At the end of the 12-week treatment period, anti-PCSK9 vaccine-related macroscopic findings at the intramuscular injection sites (red discoloration and accumulation of tan or white material) occurred at both the 100 and 600 µg doses, which corresponded microscopically to degeneration/necrosis of skeletal muscle. Accumulation of tan material at the injection site which correlated microscopically to degeneration/necrosis of skeletal muscle was also seen in the animals treated with the adjuvant control; however, the severity of these lesions was less than seen in the vaccine-treated animals (*i.e.,* the anti-PCSK9 vaccine exacerbated the adjuvant-related lesions). Both the macroscopic and microscopic lesions were still present at both the 100 and 600 µg doses at the end of the four-week recovery period, as well as in the animals treated with the adjuvant control.

Based on the lack of any evidence of systemic toxicity, the No-Observed-Adverse-Effect Level (NOAEL) in this study was the 600 µg dose.
